# Supplementary material for: Mapping stakeholders’ relationships management in fulfilling corporate social responsibility: A study of China’s construction industry
Source: PLoS One. 2024 Jan 5;19(1):e0294588. doi: 10.1371/journal.pone.0294588 (PMC10769051; doi:10.1371/journal.pone.0294588)
Supplement: S1 Table — (DOCX) [file pone.0294588.s002.docx]

# S-Table 1. Categorization of CSR in Construction.

| **References** | **Title** | **Country** | **CSR Categorization description** |
| --- | --- | --- | --- |
| [Jones et al. (2006) [1]](#_ENREF_1) | Corporate social responsibility and the UK construction industry | UK | CSR practices can be categorized into environment; health and safety; human resources; supply chain management; customers and communities; and governance and ethics. |
| [Petrovic‐Lazarevic (2008) [2]](#_ENREF_2) | The development of corporate social responsibility in the Australian construction industry | Australian | CSR can be categorized into corporate governance structure and CSR, organizational vision statement, healthy working environment, application of ISO 14001 EMS and OHS measures, communication of values and ethics. |
| [Brown et al. (2009) [3]](#_ENREF_3) | Corporate responsibility reporting in UK construction | UK | Topics from CSR reports include community involvement, socially responsible production processes, socially responsible employee relations. |
| [Barthorpe (2010) [4]](#_ENREF_4) | Implementing corporate social responsibility in the UK construction industry | UK | Types of CSR activities include considerate, safe, environmentally aware, respectful, good neighbor, responsible, clean, and accountable. |
| [Zhao et al. (2012) [5]](#_ENREF_5) | A corporate social responsibility indicator system for construction enterprises | China | Includes responsibilities to the following 11 categories of stakeholders: employees, customers, shareholders, creditors, suppliers and partners, environment and resources agencies, local communities, government, competitors and NGOs. |
| [Huang et al. (2012) [6]](#_ENREF_6) | An empirical analysis of the influences of corporate social responsibility on organizational performance of Taiwan’s construction industry: using corporate image as a mediator | Taiwan | The CSR includes resource conservation, pollution prevention, social commitment, and social participation. |
| [Yam (2013) [7]](#_ENREF_7) | The practice of corporate social responsibility by Malaysian developers | Malaysian | CSR practices include four dimensions: community, environment, workplace, and marketplace. |
| [Wuttke et al. (2014) [8]](#_ENREF_8) | Poverty alleviation through CSR in the Indian construction industry | Indian | Three types of activities in the CSR model: core business orientation, philanthropy, and political participation and advocacy. |
| [Bevan et al. (2015) [9]](#_ENREF_9) | Implementation of corporate social responsibility in Australian construction SMEs | Australian | CSR practices are categorized into: environmental aspect, social aspect, and ethical aspect. |
| [Liao et al. (2015) [10]](#_ENREF_10) | Selection of the approach for producing a weighting scheme for the CSR evaluation framework | China | The authors propose process-based CSR for the construction industry, including labor practices, environment, fair operating practices, community involvement and development, human rights, human rights, and organizational governance. |
| [Zeng et al. (2015) [11]](#_ENREF_11) | Social responsibility of major infrastructure projects in China | China | Propose social, economic, legal and ethical CSR from different stages and different stakeholders. |
| [Wu et al. (2015) [12]](#_ENREF_12) | Perception of corporate social responsibility: the case of Chinese international contractors | China | Propose 8 aspects of CSR from the perspective of stakeholders: employees, clients, business partners, competitors, local communities, governments, shareholders, and the natural environment. |
| [Wu et al. (2015) [13]](#_ENREF_13) | Benchmarking analysis on corporate social responsibility perception of Chinese international contractors |  |  |
| [Zhao et al. (2016) [14]](#_ENREF_14) | Corporate social responsibility for construction contractors: a China study | China | From a stakeholder perspective, CSR is discussed at the project level and at the organizational level. |
| [Jiang et al. (2016) [15]](#_ENREF_15) | Key activity areas of corporate social responsibility (CSR) in the construction industry: a study of China | China | CSR activities can be condensed into six key factors or key areas: environment preservation, construction quality and safety, well-being of local community, employees’ interests, clients’ interests, CSR institutional arrangement. |
| [Lu et al. (2016) [16]](#_ENREF_16) | Corporate Social Responsibility Disclosures in International Construction Business: Trends and Prospects | General | Six CSR performance indicators are proposed, including environment, society, labor practices and human rights, product responsibility, information transparency, and economic indicators. |
| [Lin et al. (2017) [17]](#_ENREF_17) | Who should take the responsibility? Stakeholders' power over social responsibility issues in construction projects | General | The social responsibilities of project fall in three project lifecycle stages and seven dimensions: 1) organizational government, 2) human rights, 3) labor protection, 4) environment, 5) fair operation, 6) customer issues and 7) community involvement and development. |
| [Lin et al. (2017) [18]](#_ENREF_18) | An indicator system for evaluating megaproject social responsibility | China | Consider the social responsibility of giant projects from the project and organizational level in combination with the perspective of stakeholders. It includes: economic responsibility, legal and regulatory responsibility, ethical and environmental responsibility, political responsibility, and social responsibility strategy. |
| [Loosemore et al. (2017) [19]](#_ENREF_19) | Linking corporate social responsibility and organizational performance in the construction industry | Australia and New Zealand | CSR focus, workplace strategies, supply chain strategies, community engagement strategies, environmental strategies, perceived benefits of CSR. |
| [Liao et al. (2017) [20]](#_ENREF_20) | Communicating the corporate social responsibility (CSR) of international contractors: Content analysis of CSR reporting | General (Asia, EU, US/Canada, and China) | These dimensions include labor practices, the environment, fair operating practices, community involvement and development, human rights, shareholders’ rights, and organizational governance. |
| [Liao et al. (2018) [21]](#_ENREF_21) | Does corporate social performance pay back quickly? A longitudinal content analysis on international contractors | General (Asia, EU, US/Canada, and China) |  |
| [Loosemore et al. (2018) [22]](#_ENREF_22) | A comparison of corporate social responsibility practices in the Singapore, Australia and New Zealand construction industries | Singapore, Australia and New Zealand | CSR leadership, vision and mission; CSR focus; environmental strategies; workplace strategies; supply chain strategies; community engagement strategies. |
| [Loosemore et al. (2018) [23]](#_ENREF_23) | Mapping corporate social responsibility strategies in the construction and engineering industry | Australia and New Zealand |  |
| [Gao-Zeller et al. (2019) [24]](#_ENREF_24) | Driving Mechanism of CSR Strategy in Chinese Construction Companies based on Neo-Institutional Theory | China | The CSR strategies for huge Chinese construction corporations (HCCCs) can be divided into four categories: environmental protection, fair competition, community participation and employee protection. |
| [Li et al. (2019) [25]](#_ENREF_25) | Institutional pressures on corporate social responsibility strategy in construction corporations: The role of internal motivations | China | CSR strategies scale for huge Chinese construction corporations (HCCCs) includes four dimensions (i.e., environmental protection, fair competition, community participation, and employee protection) and 23 items. |
| [Zhang et al. (2019) [26]](#_ENREF_26) | Drivers, motivations, and barriers to the implementation of corporate social responsibility practices by construction enterprises： A review | General | The practices of CSR include: shareholder interests, environment preservation, well-being of local community, employee interests, customer interests, supplier or partner interests, government commitment, fair operation and competition, NGO practices, and CSR institutional arrangement. |
| [Xie et al. (2020) [27]](#_ENREF_27) | Understanding the CSR awareness of large construction enterprises in China | China | Corporate governance, environmental management, employee health and safety, economic responsibility, community engagement. |
| [Velychko et al. (2020) [28]](#_ENREF_28) | Corporate social responsibility in the system of interaction between stakeholders of construction enterprises | General | The indicators of CSR assessment are formed, considering the level of interaction with stakeholders, including 30 items. |
| [Ye et al. (2020) [29]](#_ENREF_29) | Corporate social responsibility “glocalisation”: Evidence from the international construction business | General | Economic initiatives, labor practice, product responsibility, social initiatives, human rights, environmental initiatives. |
| [Wang et al. (2020) [30]](#_ENREF_30) | Developing a corporate social responsibility framework for sustainable construction using partial least squares structural equation modeling | General | The conceptual CSR framework is based on the five aspects of sustainable construction: economic, environmental, social, stakeholders, and health and safety. |
| [Guo et al. (2021) [31]](#_ENREF_31) | The inverse U-shaped relationship between corporate social responsibility and competitiveness: Evidence from Chinese international construction companies | China | Five categories to evaluate a corporate social performance: 1) shareholders, 2) employees, 3) suppliers, clients, and consumers, 4) environment, and 5) community. |
| [Zhang et al. (2021) [32]](#_ENREF_32) | Mapping Perceptions and Implementation of Corporate Social Responsibility for Construction Firms via Importance–Performance Analysis: Paths of Improvement | China | Construction firms’ CSR practices can be conceptualized and categorized into nine different dimensions: 1) shareholders’ interests; 2) environmental preservation; 3) the well-being of local communities and the public; 4) customers’ interests; 5) employees’ interests; 6) suppliers’ and partners’ interests; 7) government commitment and industry development; 8) fair operation and competition; and 9) CSR institutional arrangement. |
| [Zhang et al. (2022) [33]](#_ENREF_33) | Key practices and impact factors of corporate social responsibility implementation: Evidence from construction firms | China |  |
| [Zhang et al. (2022) [34]](#_ENREF_34) | Modeling influence mechanism of factors on corporate social responsibility implementation: evidence from Chinese construction firms | China |  |
| [Zhang et al. (2022) [35]](#_ENREF_35) | Linking corporate social responsibility (CSR) practices and organizational performance in the construction industry: A resource collaboration network | China |  |
| [Zhang et al. (2022) [36]](#_ENREF_36) | Unveiling corporate social responsibility awareness and implementation: a study of the Chinese construction firms | China |  |
| [Nguyen (2023) [37]](#_ENREF_37) | Investigating the relationship between corporate social responsibility implementation and contractor competitiveness | Vietnam | Eight key stakeholders can be categorized as CSR, with 30 measurable indicators, including customer, government, employee, shareholder, competitor, supplier and partner, society, and environment. |
| [Nguyen (2022) [38]](#_ENREF_38) | A corporate social responsibility implementation index for architectural design firms in Vietnam | Vietnam | 25 CSR activities for architectural design firms, including clients’ interest, employees’ interest, community engagement, ethics CSR, shareholders and partners’ interest, environment preservation. |

# References

1. Jones P, Comfort D, Hillier D. Corporate social responsibility and the UK construction industry. Journal of Corporate Real Estate. 2006; 8(3): 134-150.

2. Petrovic‐Lazarevic S. The development of corporate social responsibility in the Australian construction industry. Construction Management and Economics. 2008; 26(2): 93-101.

3. Brown J, Parry T, Moon J. Corporate responsibility reporting in UK construction. Proceedings of the Institution of Civil Engineers - Engineering Sustainability. 2009; 162(4): 193-205.

4. Barthorpe S. Implementing corporate social responsibility in the UK construction industry. Property Management. 2010; 28(1): 4-17.

5. Zhao Z Y, Zhao X J, Davidson K, Zuo J. A corporate social responsibility indicator system for construction enterprises. Journal of Cleaner Production. 2012; 29-30: 277-289.

6. Huang C-F, Lien H-C. An empirical analysis of the influences of corporate social responsibility on organizational performance of Taiwan’s construction industry: using corporate image as a mediator. Construction Management and Economics. 2012; 30(4): 263-275.

7. Yam S. The practice of corporate social responsibility by Malaysian developers. Property Management. 2013; 31(1): 76-91.

8. Wuttke M, Vilks A. Poverty alleviation through CSR in the Indian construction industry. Journal of Management Development. 2014; 33(2): 119-130.

9. Bevan E a M, Yung P. Implementation of corporate social responsibility in Australian construction SMEs. Engineering, Construction and Architectural Management. 2015; 22(3): 295-311.

10. Liao P C, Xue J, Liu B, Fang D. Selection of the approach for producing a weighting scheme for the CSR evaluation framework. KSCE Journal of Civil Engineering. 2015; 19(6): 1549-1559.

11. Zeng S X, Ma H Y, Lin H, Zeng R C, Tam V W Y. Social responsibility of major infrastructure projects in China. International Journal of Project Management. 2015; 33(3): 537-548.

12. Wu C L, Fang D P, Liao P C, Xue J W, Li Y, Wang T. Perception of corporate social responsibility: the case of Chinese international contractors. Journal of Cleaner Production. 2015; 107: 185-194.

13. Wu C L, Yeh J L, Liao P C, Fang D P. Benchmarking analysis on corporate social responsibility perception of Chinese international contractors. Journal of the Chinese Institute of Civil and Hydraulic Engineering. 2015; 27(1): 75-80.

14. Zhao Z-Y, Zhao X-J, Zuo J, Zillante G. Corporate social responsibility for construction contractors: a China study. Journal of Engineering, Design and Technology. 2016; 14(3): 614-640.

15. Jiang W, Wong J K W. Key activity areas of corporate social responsibility (CSR) in the construction industry: a study of China. Journal of Cleaner Production. 2016; 113: 850-860.

16. Lu W, Ye M, Flanagan R, Ye K. Corporate Social Responsibility Disclosures in International Construction Business: Trends and Prospects. Journal of Construction Engineering and Management. 2016; 142(1): 04015053.

17. Lin X, Ho C M F, Shen G Q P. Who should take the responsibility? Stakeholders' power over social responsibility issues in construction projects. Journal of Cleaner Production. 2017; 154: 318-329.

18. Lin H, Zeng S, Ma H, Zeng R, Tam V W Y. An indicator system for evaluating megaproject social responsibility. International Journal of Project Management. 2017; 35(7): 1415-1426.

19. Loosemore M, Lim B T H. Linking corporate social responsibility and organizational performance in the construction industry. Construction Management and Economics. 2017; 35(3): 90-105.

20. Liao P C, Xia N N, Wu C L, Zhang X L, Yeh J L. Communicating the corporate social responsibility (CSR) of international contractors: Content analysis of CSR reporting. Journal of Cleaner Production. 2017; 156: 327-336.

21. Liao P-C, Shih Y-N, Wu C-L, Zhang X-L, Wang Y. Does corporate social performance pay back quickly? A longitudinal content analysis on international contractors. Journal of Cleaner Production. 2018; 170: 1328-1337.

22. Loosemore M, Lim B T H, Ling F Y Y, Zeng H Y. A comparison of corporate social responsibility practices in the Singapore, Australia and New Zealand construction industries. Journal of Cleaner Production. 2018; 190: 149-159.

23. Loosemore M, Lim B T H. Mapping corporate social responsibility strategies in the construction and engineering industry. Construction Management and Economics. 2018; 36(2): 67-82.

24. Gao-Zeller X J, Li X D, Yang F, Zhu W N. Driving Mechanism of CSR Strategy in Chinese Construction Companies based on Neo-Institutional Theory. KSCE JOURNAL OF CIVIL ENGINEERING. 2019; 23(5): 1939-1951.

25. Li X, Gao-Zeller X, Rizzuto T E, Yang F. Institutional pressures on corporate social responsibility strategy in construction corporations: The role of internal motivations. Corporate Social Responsibility and Environmental Management. 2019; 26(4): 721-740.

26. Zhang Q, Oo B L, Lim B T H. Drivers, motivations, and barriers to the implementation of corporate social responsibility practices by construction enterprise: A review. Journal of Cleaner Production. 2019; 210: 563-584.

27. Xie L, Xu T, Le Y, Chen Q, Xia B, Skitmore M. Understanding the CSR awareness of large construction enterprises in China. Advances in Civil Engineering. 2020; 2020: 8866511.

28. Velychko V, Prunenko D, Grytskov E. Corporate Social Responsibility in The System of Interaction Between Stakeholders of Construction Enterprises. Baltic Journal of Economic Studies. 2020; 6(5): 64-72.

29. Ye M, Lu W, Flanagan R, Chau K W. Corporate social responsibility 'glocalisation': Evidence from the international construction business. Corporate Social Responsibility & Environmental Management. 2020; 27(2): 655-669.

30. Wang L, Zhang P, Ma L, Cong X, Skibniewski M J. Developing a corporate social responsibility framework for sustainable construction using partial least squares structural equation modeling. Technological and Economic Development of Economy. 2020; 26(1): 186-212.

31. Guo H, Lu W. The inverse U-shaped relationship between corporate social responsibility and competitiveness: Evidence from Chinese international construction companies. Journal of Cleaner Production. 2021; 295: 126374.

32. Zhang Q, Lan Oo B, Lim Benson Teck H. Mapping Perceptions and Implementation of Corporate Social Responsibility for Construction Firms via Importance–Performance Analysis: Paths of Improvement. Journal of Management in Engineering. 2021; 37(6): 04021061.

33. Zhang Q, Oo B L, Lim B T-H. Key practices and impact factors of corporate social responsibility implementation: Evidence from construction firms. Engineering, Construction and Architectural Management. 2022; ahead-of-print(ahead-of-print).

34. Zhang Q, Oo B L, Lim B T-H. Modeling influence mechanism of factors on corporate social responsibility implementation: evidence from Chinese construction firms. Engineering, Construction and Architectural Management. 2022; ahead-of-print(ahead-of-print).

35. Zhang Q, Oo B L, Lim B T H. Linking corporate social responsibility (CSR) practices and organizational performance in the construction industry: A resource collaboration network. Resources, Conservation and Recycling. 2022; 179: 106113.

36. Zhang Q, Oo B L, Lim B T H. Unveiling corporate social responsibility awareness and implementation: a study of the Chinese construction firms. Journal of Environmental Planning and Management. 2022: 1-29.

37. Nguyen M V. Investigating the relationship between corporate social responsibility implementation and contractor competitiveness. Engineering, Construction and Architectural Management. 2023; ahead-of-print(ahead-of-print).

38. Nguyen M V. Corporate social responsibility performance and its effects on climate for innovation and opportunism: evidence from Vietnamese architectural design firms. Engineering, Construction and Architectural Management. 2022.
