# Supplementary material for: Mapping stakeholders’ relationships management in fulfilling corporate social responsibility: A study of China’s construction industry
Source: PLoS One. 2024 Jan 5;19(1):e0294588. doi: 10.1371/journal.pone.0294588 (PMC10769051; doi:10.1371/journal.pone.0294588)
Supplement: S2 Table — (DOCX) [file pone.0294588.s003.docx]

# S-Table 2. A full-preliminary list of CSR activities.

| Codes | Behaviors | CSR report tools | Literature |
| --- | --- | --- | --- |
| • Management of Responsibility (R) is the management of corporate social responsibility itself, including the planning, formulation, and implementation of social responsibility activities, establishment and organization of relevant departments, etc., to ensure the effective operation of CSR behaviors [[1](#_ENREF_1)]. In this way, CSR becomes part of an organization’s vision, mission or policy [[2](#_ENREF_2)], an integral part of company operations [[3](#_ENREF_3)], and provides an effective management of social responsibility provides a platform [[4](#_ENREF_4)]. | | | |
| R01 | Develop CSR plans | √ | [Liao et al. (2017) [5]](#_ENREF_5), [Loosemore et al. (2018) [6]](#_ENREF_6), [Lin et al. (2017) [7]](#_ENREF_7), [Xie et al. (2020) [8]](#_ENREF_8), [Zhang et al. (2022) [9]](#_ENREF_9), [Zhang et al. (2021) [10]](#_ENREF_10), [Wu et al. (2015) [11]](#_ENREF_11), [Petrovic‐Lazarevic (2008) [12]](#_ENREF_12), [Liao et al. (2015) [13]](#_ENREF_13) |
| R02 | Promote the integration of CSR into corporate strategies and routine | √ | [Loosemore et al. (2018) [6]](#_ENREF_6), [Sabini et al. (2019) [14]](#_ENREF_14), [Martens et al. (2016) [15]](#_ENREF_15), [Silvius (2016) [16]](#_ENREF_16), [Loosemore et al. (2018) [17]](#_ENREF_17) |
| R03 | Guide managers to participate in CSR· | √ | [Loosemore et al. (2018) [6]](#_ENREF_6), [Loosemore et al. (2018) [17]](#_ENREF_17), [Wang et al. (2020) [18]](#_ENREF_18), [Petrovic‐Lazarevic (2008) [12]](#_ENREF_12) |
| R04 | Establish CSR leadership bureau and mechanisms | √ | [Liao et al. (2017) [5]](#_ENREF_5), [Loosemore et al. (2018) [6]](#_ENREF_6) |
| R05 | Make CSR management systems and set up departments | √ | [Liao et al. (2017) [5]](#_ENREF_5), [Jiang et al. (2016) [19]](#_ENREF_19), [Loosemore et al. (2018) [17]](#_ENREF_17) , [Lin et al. (2017) [7]](#_ENREF_7) |
| R06 | Develop/organize CSR training | √ | [Liao et al. (2017) [5]](#_ENREF_5), [Zhang et al. (2022) [9]](#_ENREF_9) |
| R07 | Participate in the making of CSR standards and initiatives | √ |  |
| R08 | Establish communication mechanisms | √ | [Zhang et al. (2022) [9]](#_ENREF_9) |
| R09 | Join CSR organization/convention | √ | [Jiang et al. (2016) [19]](#_ENREF_19) |
| R10 | CSR crisis management |  | [Loosemore et al. (2018) [6]](#_ENREF_6) |
| R11 | CSR third-party assessment | √ | [Loosemore et al. (2018) [17]](#_ENREF_17) |
| • Corporate Governance (G) integrates CSR behaviors into the day-to-day operations and management of an enterprise and is a responsibility to the entire enterprise to determine the wide range of uses for which organizational resources will be deployed to the company and to resolve conflicts among the countless players in the organization [[20](#_ENREF_20)]. | | | |
| G01 | Improve corporate governance structure | √ | [Maignan et al. (2000) [21]](#_ENREF_21), [Gorden et al. (1992) [22]](#_ENREF_22), [Manowong et al. (2006) [23]](#_ENREF_23), [Oppong et al. (2017) [24]](#_ENREF_24), [Xie et al. (2020) [8]](#_ENREF_8), [Jones et al. (2006) [25]](#_ENREF_25) |
| G02 | Invest resources to innovate the corporate management |  | [Loosemore et al. (2018) [6]](#_ENREF_6), [Wang et al. (2020) [18]](#_ENREF_18), [Maignan et al. (2000) [21]](#_ENREF_21), [Turker (2009) [26]](#_ENREF_26), [Zhang et al. (2022) [9]](#_ENREF_9), [Jones et al. (2006) [25]](#_ENREF_25) |
| G03 | Legal management | √ | [Lin et al. (2017) [7]](#_ENREF_7), [Zhao et al. (2016) [27]](#_ENREF_27), [Loosemore et al. (2018) [17]](#_ENREF_17), [Xie et al. (2020) [8]](#_ENREF_8), [Zhang et al. (2022) [9]](#_ENREF_9), [Zhao et al. (2012) [28]](#_ENREF_28), [Zhang et al. (2019) [29]](#_ENREF_29), [Yam (2013) [30]](#_ENREF_30), [Nguyen (2023) [31]](#_ENREF_31), Nguyen and Ha (2023) |
| G04 | Maintain investor relations and protect shareholder rights to help them increase revenue and engage in decision-making, and enhancing communication and relationship management among them. | √ | [Liao et al. (2017) [5]](#_ENREF_5), [Wang et al. (2020) [18]](#_ENREF_18), [Lin et al. (2017) [7]](#_ENREF_7), [Zhao et al. (2016) [27]](#_ENREF_27), [Zhang et al. (2022) [9]](#_ENREF_9), [Zhao et al. (2012) [28]](#_ENREF_28), [Zhang et al. (2019) [29]](#_ENREF_29), [Jones et al. (2006) [25]](#_ENREF_25), [Liao et al. (2015) [13]](#_ENREF_13), [Nguyen (2023) [31]](#_ENREF_31), Nguyen and Ha (2023) |
| G05 | Disclosure information to improve transparency | √ | [Liao et al. (2017) [5]](#_ENREF_5), [Loosemore et al. (2018) [6]](#_ENREF_6), [Lin et al. (2017) [7]](#_ENREF_7), [Zhao et al. (2016) [27]](#_ENREF_27), [Xie et al. (2020) [8]](#_ENREF_8), [Zhang et al. (2022) [9]](#_ENREF_9), [Wu et al. (2015) [11]](#_ENREF_11), [Zeng et al. (2015) [32]](#_ENREF_32), [Lin et al. (2017) [33]](#_ENREF_33), [Zhang et al. (2019) [29]](#_ENREF_29), [Lu et al. (2016) [34]](#_ENREF_34), [Liao et al. (2015) [13]](#_ENREF_13), [Nguyen (2023) [31]](#_ENREF_31), Nguyen and Ha (2023) |
| G06 | Prohibition of bribery and corruption | √ | [Liao et al. (2017) [5]](#_ENREF_5), [Loosemore et al. (2018) [6]](#_ENREF_6), [Martens et al. (2017) [35]](#_ENREF_35), [Darko et al. (2016) [36]](#_ENREF_36), [Zhao et al. (2016) [27]](#_ENREF_27), [Loosemore et al. (2018) [17]](#_ENREF_17), [Xie et al. (2020) [8]](#_ENREF_8), [Zhang et al. (2022) [9]](#_ENREF_9), [Wu et al. (2015) [11]](#_ENREF_11), [Bevan et al. (2015) [37]](#_ENREF_37), [Zeng et al. (2015) [32]](#_ENREF_32), [Lin et al. (2017) [7]](#_ENREF_7), [Lin et al. (2017) [33]](#_ENREF_33), [Loosemore et al. (2017) [38]](#_ENREF_38), [Brown et al. (2009) [39]](#_ENREF_39), [Guo et al. (2021) [40]](#_ENREF_40), [Lu et al. (2016) [34]](#_ENREF_34), [Nguyen (2023) [31]](#_ENREF_31), Nguyen and Ha (2023) |
| G07 | Establish operating mechanism to improve the profit | √ | [Wang et al. (2020) [18]](#_ENREF_18) |
| G08 | Promote corporate brand |  | [Loosemore et al. (2018) [6]](#_ENREF_6), [Loosemore et al. (2017) [38]](#_ENREF_38) |
| • Safe Construction and Quality (Q) is the basic requirement of construction behavior, including maintaining and improving the safety and quality of construction, reflecting the ability of construction units to safely deliver high-quality products [[19](#_ENREF_19)] and is an important aspect of CSR practices in the industry [[11](#_ENREF_11)]. | | | |
| Q01 | Acquire quality management certification | √ | [Loosemore et al. (2018) [6]](#_ENREF_6), [Jiang et al. (2016) [19]](#_ENREF_19), [Maignan et al. (2000) [21]](#_ENREF_21), [Oppong et al. (2017) [24]](#_ENREF_24), [Zhang et al. (2022) [9]](#_ENREF_9), [Guo et al. (2021) [40]](#_ENREF_40) |
| Q02 | Optimize product/service quality management system | √ | [Wang et al. (2020) [18]](#_ENREF_18), [Lin et al. (2017) [7]](#_ENREF_7), [Zhao et al. (2016) [27]](#_ENREF_27), [Zhang et al. (2022) [9]](#_ENREF_9), [Zhao et al. (2012) [28]](#_ENREF_28), [Zhang et al. (2019) [29]](#_ENREF_29), [Petrovic‐Lazarevic (2008) [12]](#_ENREF_12), [Guo et al. (2021) [40]](#_ENREF_40) |
| Q03 | Establish an R&D and innovation system | √ | [Wang et al. (2020) [18]](#_ENREF_18), [Zhao et al. (2012) [28]](#_ENREF_28), [Zhao et al. (2016) [27]](#_ENREF_27), [Zhang et al. (2022) [9]](#_ENREF_9), [Lin et al. (2017) [7]](#_ENREF_7), [Zhang et al. (2019) [29]](#_ENREF_29) |
| Q04 | Develop customer-oriented product | √ | [Zhao et al. (2016) [27]](#_ENREF_27), [Wu et al. (2015) [11]](#_ENREF_11), [Zhao et al. (2012) [28]](#_ENREF_28), [Lin et al. (2017) [33]](#_ENREF_33), [Gao-Zeller et al. (2019) [41]](#_ENREF_41), [Li et al. (2019) [42]](#_ENREF_42), [Liao et al. (2015) [13]](#_ENREF_13), [Nguyen (2023) [31]](#_ENREF_31) |
| Q05 | Provide customers with detailed product warnings and inform customers of potential risks and threats | √ | [Zhao et al. (2012) [28]](#_ENREF_28), [Zhao et al. (2016) [27]](#_ENREF_27), [Liao et al. (2015) [13]](#_ENREF_13) |
| Q06 | Ensure fair product/service transaction | √ | [Zhao et al. (2016) [27]](#_ENREF_27), [Zhang et al. (2022) [9]](#_ENREF_9), [Bevan et al. (2015) [37]](#_ENREF_37), [Jones et al. (2006) [25]](#_ENREF_25), [Huang et al. (2012) [43]](#_ENREF_43), [Nguyen (2023) [31]](#_ENREF_31) |
| Q07 | Advocate sustainable consumption | √ | [Jiang et al. (2016) [19]](#_ENREF_19), [Zhang et al. (2022) [9]](#_ENREF_9), [Velychko et al. (2020) [44]](#_ENREF_44) |
| Q08 | Protect consumers’ privacy | √ | [Jiang et al. (2016) [19]](#_ENREF_19), [Loosemore et al. (2018) [17]](#_ENREF_17), [Zhang et al. (2022) [9]](#_ENREF_9), [Bevan et al. (2015) [37]](#_ENREF_37), [Brown et al. (2009) [39]](#_ENREF_39), [Velychko et al. (2020) [44]](#_ENREF_44) |
| Q09 | Establish/improve the after-sales service system | √ | [Jiang et al. (2016) [19]](#_ENREF_19), [Zhao et al. (2012) [28]](#_ENREF_28), [Zhao et al. (2016) [27]](#_ENREF_27), [Zhang et al. (2022) [9]](#_ENREF_9), [Bevan et al. (2015) [37]](#_ENREF_37), [Lin et al. (2017) [33]](#_ENREF_33), [Jones et al. (2006) [25]](#_ENREF_25), [Brown et al. (2009) [39]](#_ENREF_39), [Guo et al. (2021) [40]](#_ENREF_40), [Nguyen (2023) [31]](#_ENREF_31), Nguyen and Ha (2023) |
| Q10 | Implement/optimize quality training | √ | [Xie et al. (2020) [8]](#_ENREF_8), [Zhang et al. (2022) [9]](#_ENREF_9), [Wu et al. (2015) [11]](#_ENREF_11), [Zeng et al. (2015) [32]](#_ENREF_32), [Lin et al. (2017) [7]](#_ENREF_7), [Zhang et al. (2019) [29]](#_ENREF_29), [Jones et al. (2006) [25]](#_ENREF_25), [Yam (2013) [30]](#_ENREF_30), [Wuttke et al. (2014) [45]](#_ENREF_45), [Ye et al. (2020) [46]](#_ENREF_46), [Brown et al. (2009) [39]](#_ENREF_39), [Guo et al. (2021) [40]](#_ENREF_40), [Lu et al. (2016) [34]](#_ENREF_34), [Li et al. (2019) [42]](#_ENREF_42), [Liao et al. (2015) [13]](#_ENREF_13), [Nguyen (2023) [31]](#_ENREF_31), Nguyen and Ha (2023) |
| Q11 | Employee safety education and training | √ | [Jiang et al. (2016) [19]](#_ENREF_19), [Zhao et al. (2016) [27]](#_ENREF_27), [Xie et al. (2020) [8]](#_ENREF_8), [Zhang et al. (2022) [9]](#_ENREF_9), [Wu et al. (2015) [11]](#_ENREF_11), [Zhao et al. (2012) [28]](#_ENREF_28), [Zeng et al. (2015) [32]](#_ENREF_32), [Lin et al. (2017) [7]](#_ENREF_7), [Zhang et al. (2019) [29]](#_ENREF_29), [Jones et al. (2006) [25]](#_ENREF_25), [Yam (2013) [30]](#_ENREF_30), [Wuttke et al. (2014) [45]](#_ENREF_45), [Ye et al. (2020) [46]](#_ENREF_46), [Brown et al. (2009) [39]](#_ENREF_39), [Guo et al. (2021) [40]](#_ENREF_40), [Lu et al. (2016) [34]](#_ENREF_34), [Li et al. (2019) [42]](#_ENREF_42), [Liao et al. (2015) [13]](#_ENREF_13), [Nguyen (2023) [31]](#_ENREF_31), Nguyen and Ha (2023) |
| Q12 | Implement/optimize safety production management system | √ | [Jiang et al. (2016) [19]](#_ENREF_19), [Xie et al. (2020) [8]](#_ENREF_8), [Zhang et al. (2022) [9]](#_ENREF_9), [Zhao et al. (2012) [28]](#_ENREF_28), [Brown et al. (2009) [39]](#_ENREF_39) |
| Q13 | Application/optimization of security management prevention mechanism | √ | [Wang et al. (2020) [18]](#_ENREF_18) |
| Q14 | Implement safety responsibility management |  | [Jiang et al. (2016) [19]](#_ENREF_19), [Oppong et al. (2017) [24]](#_ENREF_24), [Zhang et al. (2022) [9]](#_ENREF_9), [Jones et al. (2006) [25]](#_ENREF_25), [Guo et al. (2021) [40]](#_ENREF_40), [Liao et al. (2015) [13]](#_ENREF_13) |
| Q15 | Establish regular communication mechanisms with customers | √ | [Zhao et al. (2016) [27]](#_ENREF_27), [Jones et al. (2006) [25]](#_ENREF_25), [Nguyen (2023) [31]](#_ENREF_31) |
| • Good Partnership (P) is mainly responsible for suppliers, competitors, and other associations and alliances. A responsible construction company should be able to develop a better relationship with its supply chain and partnerships [[47](#_ENREF_47)], to ensure they are adequately paid. | | | |
| P01 | Implement/apply supply chain management mechanism | √ | [Jiang et al. (2016) [19]](#_ENREF_19), [Ye et al. (2020) [46]](#_ENREF_46), [Nguyen (2023) [31]](#_ENREF_31) |
| P02 | Carry out supplier CSR training | √ | [Jiang et al. (2016) [19]](#_ENREF_19), [Oppong et al. (2017) [24]](#_ENREF_24), [Zhang et al. (2022) [9]](#_ENREF_9) |
| P03 | Establish a partner communication mechanism to improve cooperation | √ | [Wang et al. (2020) [18]](#_ENREF_18), [Zhao et al. (2012) [28]](#_ENREF_28), [Zhao et al. (2016) [27]](#_ENREF_27), [Loosemore et al. (2018) [17]](#_ENREF_17), [Zhang et al. (2022) [9]](#_ENREF_9), [Wu et al. (2015) [11]](#_ENREF_11), [Bevan et al. (2015) [37]](#_ENREF_37), [Zhang et al. (2019) [29]](#_ENREF_29), [Jones et al. (2006) [25]](#_ENREF_25), [Gao-Zeller et al. (2019) [41]](#_ENREF_41), [Li et al. (2019) [42]](#_ENREF_42), [Liao et al. (2015) [13]](#_ENREF_13), [Nguyen (2023) [31]](#_ENREF_31), Nguyen and Ha (2023) |
| P04 | Protect intellectual property | √ | [Loosemore et al. (2018) [6]](#_ENREF_6), [Zhang et al. (2019) [29]](#_ENREF_29) |
| P05 | Communication within the industry to promote industry development | √ | [Loosemore et al. (2018) [6]](#_ENREF_6) |
| P06 | Alliance with other companies, institutions, groups, etc. | √ |  |
| P07 | Conducting green procurement | √ | [Loosemore et al. (2018) [6]](#_ENREF_6), [Loosemore et al. (2018) [17]](#_ENREF_17), [Zhang et al. (2022) [9]](#_ENREF_9), [Wang et al. (2020) [18]](#_ENREF_18), [Jiang et al. (2016) [19]](#_ENREF_19), [Bevan et al. (2015) [37]](#_ENREF_37), [Lin et al. (2017) [33]](#_ENREF_33) |
| • Workers Interest (W) is a responsibility to employees. Employees are one of the most important stakeholders in the construction industry [[34](#_ENREF_34)]. Includes management issues such as employment policy, employment relations, workplace safety, and human development and training. | | | |
| W01 | Implement an employee career management system | √ | [Loosemore et al. (2017) [38]](#_ENREF_38), [Sabini et al. (2019) [14]](#_ENREF_14), [Silvius (2016) [16]](#_ENREF_16), [Wang et al. (2020) [18]](#_ENREF_18), [Zhao et al. (2016) [27]](#_ENREF_27), [Wu et al. (2015) [11]](#_ENREF_11), [Bevan et al. (2015) [37]](#_ENREF_37), [Loosemore et al. (2017) [38]](#_ENREF_38), [Petrovic‐Lazarevic (2008) [12]](#_ENREF_12), [Gao-Zeller et al. (2019) [41]](#_ENREF_41), [Li et al. (2019) [42]](#_ENREF_42) |
| W02 | Establish an employee promotion mechanism | √ | [Loosemore et al. (2018) [6]](#_ENREF_6), [Wang et al. (2020) [18]](#_ENREF_18), [Maignan et al. (2000) [21]](#_ENREF_21), [Zhao et al. (2016) [27]](#_ENREF_27), [Loosemore et al. (2018) [17]](#_ENREF_17), [Gao-Zeller et al. (2019) [41]](#_ENREF_41), [Li et al. (2019) [42]](#_ENREF_42), [Nguyen (2023) [31]](#_ENREF_31), Nguyen and Ha (2023) |
| W03 | Implement employer/employee relationship management | √ | [Loosemore et al. (2018) [6]](#_ENREF_6), [Jiang et al. (2016) [19]](#_ENREF_19), [Maignan et al. (2000) [21]](#_ENREF_21), [Zhao et al. (2012) [28]](#_ENREF_28), [Zhao et al. (2016) [27]](#_ENREF_27), [Loosemore et al. (2018) [17]](#_ENREF_17), [Zhang et al. (2022) [9]](#_ENREF_9), [Jones et al. (2006) [25]](#_ENREF_25), [Lu et al. (2016) [34]](#_ENREF_34), [Nguyen (2023) [31]](#_ENREF_31), Nguyen and Ha (2023) |
| W04 | Implement effective denial, report, and complaint mechanisms through the labor unions | √ | [Jiang et al. (2016) [19]](#_ENREF_19), [Zhao et al. (2012) [28]](#_ENREF_28), [Zhao et al. (2016) [27]](#_ENREF_27), [Loosemore et al. (2018) [17]](#_ENREF_17), [Zhang et al. (2022) [9]](#_ENREF_9), [Loosemore et al. (2017) [38]](#_ENREF_38), [Brown et al. (2009) [39]](#_ENREF_39), [Nguyen (2023) [31]](#_ENREF_31), Nguyen and Ha (2023) |
| W05 | Implement employee and family care plan | √ | [Loosemore et al. (2018) [6]](#_ENREF_6), [Lin et al. (2017) [7]](#_ENREF_7), [Loosemore et al. (2018) [17]](#_ENREF_17), [Zhang et al. (2022) [9]](#_ENREF_9), [Bevan et al. (2015) [37]](#_ENREF_37), [Wuttke et al. (2014) [45]](#_ENREF_45), [Brown et al. (2009) [39]](#_ENREF_39), [Li et al. (2019) [42]](#_ENREF_42), [Nguyen (2023) [31]](#_ENREF_31), Nguyen and Ha (2023) |
| W06 | Protect employees’ occupational health and safety | √ | [Loosemore et al. (2018) [17]](#_ENREF_17), [Loosemore et al. (2018) [6]](#_ENREF_6), [Martens et al. (2017) [35]](#_ENREF_35), [Wang et al. (2020) [18]](#_ENREF_18), [Zhao et al. (2016) [27]](#_ENREF_27), [Xie et al. (2020) [8]](#_ENREF_8), [Zhang et al. (2022) [9]](#_ENREF_9), [Wu et al. (2015) [11]](#_ENREF_11), [Zhao et al. (2012) [28]](#_ENREF_28), [Bevan et al. (2015) [37]](#_ENREF_37), [Zeng et al. (2015) [32]](#_ENREF_32), [Lin et al. (2017) [7]](#_ENREF_7), [Lin et al. (2017) [33]](#_ENREF_33), [Loosemore et al. (2017) [38]](#_ENREF_38), [Zhang et al. (2019) [29]](#_ENREF_29), [Petrovic‐Lazarevic (2008) [12]](#_ENREF_12), [Jones et al. (2006) [25]](#_ENREF_25), [Ye et al. (2020) [46]](#_ENREF_46), [Brown et al. (2009) [39]](#_ENREF_39), [Lu et al. (2016) [34]](#_ENREF_34), [Gao-Zeller et al. (2019) [41]](#_ENREF_41), [Liao et al. (2015) [13]](#_ENREF_13), [Nguyen (2023) [31]](#_ENREF_31), Nguyen and Ha (2023) |
| W07 | Protect the rights of migrant workers |  | [Loosemore et al. (2018) [6]](#_ENREF_6), [Maignan et al. (2000) [21]](#_ENREF_21), [Xie et al. (2020) [8]](#_ENREF_8), [Lin et al. (2017) [33]](#_ENREF_33) |
| W08 | Advocate the multicultural and individual development of employees | √ | [Liao et al. (2017) [5]](#_ENREF_5), [Loosemore et al. (2018) [6]](#_ENREF_6), [Maignan et al. (2000) [21]](#_ENREF_21), [Loosemore et al. (2018) [17]](#_ENREF_17), [Xie et al. (2020) [8]](#_ENREF_8), [Zhang et al. (2022) [9]](#_ENREF_9), [Zhao et al. (2012) [28]](#_ENREF_28), [Bevan et al. (2015) [37]](#_ENREF_37), [Loosemore et al. (2017) [38]](#_ENREF_38), [Jones et al. (2006) [25]](#_ENREF_25), [Lu et al. (2016) [34]](#_ENREF_34) |
| W09 | Protect employee privacy | √ | [Zhao et al. (2016) [27]](#_ENREF_27), [Loosemore et al. (2018) [17]](#_ENREF_17), [Zhang et al. (2022) [9]](#_ENREF_9), [Zhao et al. (2012) [28]](#_ENREF_28), [Loosemore et al. (2017) [38]](#_ENREF_38) |
| W10 | Guarantee the working environment and working conditions of employees | √ | [Wang et al. (2020) [18]](#_ENREF_18), [Turker (2009) [26]](#_ENREF_26), [Zhao et al. (2012) [28]](#_ENREF_28), [Zhao et al. (2016) [27]](#_ENREF_27), [Zhang et al. (2022) [9]](#_ENREF_9), [Wu et al. (2015) [11]](#_ENREF_11), [Petrovic‐Lazarevic (2008) [12]](#_ENREF_12), [Jones et al. (2006) [25]](#_ENREF_25), [Yam (2013) [30]](#_ENREF_30), [Wuttke et al. (2014) [45]](#_ENREF_45), [Gao-Zeller et al. (2019) [41]](#_ENREF_41), [Li et al. (2019) [42]](#_ENREF_42) |
| W11 | Sign labor contracts following the law to combat forced labor | √ | [Liao et al. (2017) [5]](#_ENREF_5), [Loosemore et al. (2017) [38]](#_ENREF_38), [Loosemore et al. (2018) [6]](#_ENREF_6), [Zhao et al. (2016) [27]](#_ENREF_27), [Zhang et al. (2022) [9]](#_ENREF_9), [Wu et al. (2015) [11]](#_ENREF_11), [Zhao et al. (2012) [28]](#_ENREF_28), [Lin et al. (2017) [33]](#_ENREF_33), [Brown et al. (2009) [39]](#_ENREF_39), [Gao-Zeller et al. (2019) [41]](#_ENREF_41), [Li et al. (2019) [42]](#_ENREF_42) |
| W12 | Prohibition of harassment and abuse of employees | √ | [Loosemore et al. (2018) [6]](#_ENREF_6), [Zhao et al. (2016) [27]](#_ENREF_27), [Loosemore et al. (2018) [17]](#_ENREF_17), [Wu et al. (2015) [11]](#_ENREF_11), [Bevan et al. (2015) [37]](#_ENREF_37), [Lin et al. (2017) [33]](#_ENREF_33), [Loosemore et al. (2017) [38]](#_ENREF_38), [Jones et al. (2006) [25]](#_ENREF_25), [Wuttke et al. (2014) [45]](#_ENREF_45), [Brown et al. (2009) [39]](#_ENREF_39), [Gao-Zeller et al. (2019) [41]](#_ENREF_41), [Li et al. (2019) [42]](#_ENREF_42) |
| W13 | Equal employment | √ | [Liao et al. (2017) [5]](#_ENREF_5), [Zhao et al. (2012) [28]](#_ENREF_28), [Zhao et al. (2016) [27]](#_ENREF_27), [Loosemore et al. (2018) [17]](#_ENREF_17), [Xie et al. (2020) [8]](#_ENREF_8), [Zhang et al. (2022) [9]](#_ENREF_9), [Wu et al. (2015) [11]](#_ENREF_11), [Bevan et al. (2015) [37]](#_ENREF_37), [Zeng et al. (2015) [32]](#_ENREF_32), [Lin et al. (2017) [33]](#_ENREF_33), [Loosemore et al. (2017) [38]](#_ENREF_38), [Jones et al. (2006) [25]](#_ENREF_25), [Yam (2013) [30]](#_ENREF_30), [Wuttke et al. (2014) [45]](#_ENREF_45), [Ye et al. (2020) [46]](#_ENREF_46), [Lu et al. (2016) [34]](#_ENREF_34), [Gao-Zeller et al. (2019) [41]](#_ENREF_41), [Li et al. (2019) [42]](#_ENREF_42), [Liao et al. (2015) [13]](#_ENREF_13), [Nguyen (2023) [31]](#_ENREF_31), Nguyen and Ha (2023) |
| W14 | Effective emergency management procedures and safety monitoring mechanisms | √ | [Wang et al. (2020) [18]](#_ENREF_18), [Zhao et al. (2012) [28]](#_ENREF_28), [Zhao et al. (2016) [27]](#_ENREF_27), [Zhang et al. (2022) [9]](#_ENREF_9), [Lin et al. (2017) [7]](#_ENREF_7), [Petrovic‐Lazarevic (2008) [12]](#_ENREF_12), [Brown et al. (2009) [39]](#_ENREF_39), [Liao et al. (2015) [13]](#_ENREF_13) |
| • Well-being of Local Community (C) is community engagement and development. On the one hand, local communities are directly affected by construction activities and face issues such as resettlement, demolition, and construction [[32](#_ENREF_32)]. On the other hand, community participation is the participation of enterprises in the development of local communities, and building a harmonious relationship with local communities helps avoid unnecessary resistance [[48](#_ENREF_48)]. The focus of community development is to effectively improve the welfare and living standards of the community through the participation of enterprises [[5](#_ENREF_5)], such as providing additional employment opportunities [[49](#_ENREF_49)], cultivating local construction talents [[48](#_ENREF_48)], etc. | | | |
| C01 | Assess public and community needs |  | [Loosemore et al. (2018) [6]](#_ENREF_6), [Sabini et al. (2019) [14]](#_ENREF_14), [Lin et al. (2017) [7]](#_ENREF_7), [Jiang et al. (2016) [19]](#_ENREF_19), [Zhao et al. (2016) [27]](#_ENREF_27), [Loosemore et al. (2018) [17]](#_ENREF_17) |
| C02 | Establish effective communication channels and participation mechanisms | √ | [Liao et al. (2017) [5]](#_ENREF_5), [Loosemore et al. (2018) [6]](#_ENREF_6), [Loosemore et al. (2018) [17]](#_ENREF_17), [Zhang et al. (2022) [9]](#_ENREF_9), [Zhao et al. (2012) [28]](#_ENREF_28), [Zeng et al. (2015) [32]](#_ENREF_32), [Loosemore et al. (2017) [38]](#_ENREF_38), [Liao et al. (2015) [13]](#_ENREF_13), [Nguyen (2023) [31]](#_ENREF_31) |
| C03 | Handle community complaints promptly |  | [Liao et al. (2017) [5]](#_ENREF_5), [Loosemore et al. (2018) [17]](#_ENREF_17), [Liao et al. (2015) [13]](#_ENREF_13) |
| C04 | Organize community vocational skills training programs and knowledge popularization activities |  | [Wang et al. (2020) [18]](#_ENREF_18), [Jiang et al. (2016) [19]](#_ENREF_19), [Zhang et al. (2022) [9]](#_ENREF_9), [Bevan et al. (2015) [37]](#_ENREF_37), [Barthorpe (2010) [50]](#_ENREF_50), [Huang et al. (2012) [43]](#_ENREF_43), [Ye et al. (2020) [46]](#_ENREF_46), [Brown et al. (2009) [39]](#_ENREF_39) |
| C05 | Priority purchase of local products and services | √ | [Jiang et al. (2016) [19]](#_ENREF_19), [Loosemore et al. (2018) [17]](#_ENREF_17), [Zhang et al. (2022) [9]](#_ENREF_9), [Bevan et al. (2015) [37]](#_ENREF_37), [Lin et al. (2017) [33]](#_ENREF_33), [Loosemore et al. (2017) [38]](#_ENREF_38), [Brown et al. (2009) [39]](#_ENREF_39), [Nguyen (2023) [31]](#_ENREF_31), Nguyen and Ha (2023) |
| C06 | Respect and protect the local culture, customs and heritage |  | [Loosemore et al. (2017) [38]](#_ENREF_38), [Zhao et al. (2016) [27]](#_ENREF_27), [Zhang et al. (2022) [9]](#_ENREF_9), [Wu et al. (2015) [11]](#_ENREF_11), [Lin et al. (2017) [33]](#_ENREF_33), [Huang et al. (2012) [43]](#_ENREF_43), [Yam (2013) [30]](#_ENREF_30), [Gao-Zeller et al. (2019) [41]](#_ENREF_41), [Li et al. (2019) [42]](#_ENREF_42), [Liao et al. (2015) [13]](#_ENREF_13) |
| C07 | Disaster prevention/relief activities |  | [Jiang et al. (2016) [19]](#_ENREF_19), [Maignan et al. (2000) [21]](#_ENREF_21), [Zhang et al. (2022) [9]](#_ENREF_9), [Zeng et al. (2015) [32]](#_ENREF_32), [Barthorpe (2010) [50]](#_ENREF_50), [Ye et al. (2020) [46]](#_ENREF_46) |
| C08 | Support the development of infrastructure and public services in local communities | √ | [Loosemore et al. (2018) [6]](#_ENREF_6), [Jiang et al. (2016) [19]](#_ENREF_19), [Zhao et al. (2012) [28]](#_ENREF_28), [Zhao et al. (2016) [27]](#_ENREF_27), [Loosemore et al. (2018) [17]](#_ENREF_17), [Zhang et al. (2022) [9]](#_ENREF_9), [Zeng et al. (2015) [32]](#_ENREF_32), [Loosemore et al. (2017) [38]](#_ENREF_38), [Yam (2013) [30]](#_ENREF_30), [Wuttke et al. (2014) [45]](#_ENREF_45), [Brown et al. (2009) [39]](#_ENREF_39), [Liao et al. (2015) [13]](#_ENREF_13) |
| C09 | Pay attention to the rights and interests of a disadvantaged group | √ | [Zhao et al. (2012) [28]](#_ENREF_28), [Zhao et al. (2016) [27]](#_ENREF_27), [Zhang et al. (2022) [9]](#_ENREF_9), [Zeng et al. (2015) [32]](#_ENREF_32), [Loosemore et al. (2017) [38]](#_ENREF_38), [Barthorpe (2010) [50]](#_ENREF_50) |
| C10 | Protect the rights of local ethnic minorities |  | [Loosemore et al. (2018) [6]](#_ENREF_6), [Zhao et al. (2016) [27]](#_ENREF_27), [Zhao et al. (2012) [28]](#_ENREF_28), [Brown et al. (2009) [39]](#_ENREF_39) |
| C11 | Alleviate community poverty | √ | [Xie et al. (2020) [8]](#_ENREF_8), [Zhang et al. (2022) [9]](#_ENREF_9), [Zeng et al. (2015) [32]](#_ENREF_32), [Yam (2013) [30]](#_ENREF_30), [Wuttke et al. (2014) [45]](#_ENREF_45), [Ye et al. (2020) [46]](#_ENREF_46) |
| C12 | Prioritize local employees | √ | [Zhao et al. (2016) [27]](#_ENREF_27), [Xie et al. (2020) [8]](#_ENREF_8), [Zhang et al. (2022) [9]](#_ENREF_9), [Wu et al. (2015) [11]](#_ENREF_11), [Zhao et al. (2012) [28]](#_ENREF_28), [Li et al. (2019) [42]](#_ENREF_42), [Liao et al. (2015) [13]](#_ENREF_13), [Velychko et al. (2020) [44]](#_ENREF_44), [Nguyen (2023) [31]](#_ENREF_31) |
| C13 | Establish a charity fund or foundation | √ | [Loosemore et al. (2018) [6]](#_ENREF_6), [Zhao et al. (2016) [27]](#_ENREF_27), [Loosemore et al. (2018) [17]](#_ENREF_17), [Wu et al. (2015) [11]](#_ENREF_11), [Zhao et al. (2012) [28]](#_ENREF_28), [Bevan et al. (2015) [37]](#_ENREF_37), [Lin et al. (2017) [7]](#_ENREF_7), [Loosemore et al. (2017) [38]](#_ENREF_38), [Barthorpe (2010) [50]](#_ENREF_50), [Huang et al. (2012) [43]](#_ENREF_43), [Yam (2013) [30]](#_ENREF_30), [Gao-Zeller et al. (2019) [41]](#_ENREF_41), [Li et al. (2019) [42]](#_ENREF_42) |
| C14 | Formulate policies and measures to support volunteer activities | √ | [Loosemore et al. (2018) [6]](#_ENREF_6), [Zhao et al. (2016) [27]](#_ENREF_27), [Zhang et al. (2022) [9]](#_ENREF_9), [Zhao et al. (2012) [28]](#_ENREF_28), [Bevan et al. (2015) [37]](#_ENREF_37), [Lin et al. (2017) [7]](#_ENREF_7), [Loosemore et al. (2017) [38]](#_ENREF_38), [Nguyen (2023) [31]](#_ENREF_31), Nguyen and Ha (2023) |
| • Environment Preservation (E) includes the attention and protection of the environment during the whole life cycle of enterprises such as corporate office, construction enterprise construction, and post-operation and maintenance of buildings. Research shows that environmental concerns have become a key criterion for the success of construction projects [[51](#_ENREF_51)]. As construction activities have a direct impact on the environment, Chinese construction practitioners advocate environmental protection [[19](#_ENREF_19)] , and environmental issues are an important aspect of the CSR agenda [[33](#_ENREF_33)]. Therefore, it is the duty of every construction enterprise to protect the environment [[52](#_ENREF_52)]. | | | |
| E01 | Establish an environmental management system, including environmental planning and management, ISO14000 certification, etc. | √ | [Loosemore et al. (2018) [6]](#_ENREF_6), [Wang et al. (2020) [18]](#_ENREF_18), [Jiang et al. (2016) [19]](#_ENREF_19), [Zhao et al. (2012) [28]](#_ENREF_28), [Loosemore et al. (2018) [17]](#_ENREF_17), [Xie et al. (2020) [8]](#_ENREF_8), [Zhang et al. (2022) [9]](#_ENREF_9), [Bevan et al. (2015) [37]](#_ENREF_37), [Lin et al. (2017) [33]](#_ENREF_33), [Loosemore et al. (2017) [38]](#_ENREF_38), [Zhang et al. (2019) [29]](#_ENREF_29), [Wuttke et al. (2014) [45]](#_ENREF_45), [Guo et al. (2021) [40]](#_ENREF_40), [Li et al. (2019) [42]](#_ENREF_42), [Nguyen (2023) [31]](#_ENREF_31) |
| E02 | Implement/optimize staff’s environmental training | √ | [Loosemore et al. (2018) [6]](#_ENREF_6), [Jiang et al. (2016) [19]](#_ENREF_19), [Zhao et al. (2012) [28]](#_ENREF_28), [Zhao et al. (2016) [27]](#_ENREF_27), [Loosemore et al. (2018) [17]](#_ENREF_17), [Xie et al. (2020) [8]](#_ENREF_8), [Zhang et al. (2022) [9]](#_ENREF_9), [Wu et al. (2015) [11]](#_ENREF_11), [Bevan et al. (2015) [37]](#_ENREF_37), [Zeng et al. (2015) [32]](#_ENREF_32), [Lin et al. (2017) [33]](#_ENREF_33), [Loosemore et al. (2017) [38]](#_ENREF_38), [Yam (2013) [30]](#_ENREF_30), [Ye et al. (2020) [46]](#_ENREF_46), [Guo et al. (2021) [40]](#_ENREF_40), [Lu et al. (2016) [34]](#_ENREF_34), [Li et al. (2019) [42]](#_ENREF_42), [Liao et al. (2015) [13]](#_ENREF_13), [Nguyen (2023) [31]](#_ENREF_31), Nguyen and Ha (2023) |
| E03 | Apply/optimize the environmental impact assessment before construction | √ | [Loosemore et al. (2018) [6]](#_ENREF_6), [Sabini et al. (2019) [14]](#_ENREF_14), [Maignan et al. (2000) [21]](#_ENREF_21), [Zhao et al. (2012) [28]](#_ENREF_28), [Wu et al. (2015) [11]](#_ENREF_11), [Bevan et al. (2015) [37]](#_ENREF_37), [Liao et al. (2015) [13]](#_ENREF_13) |
| E04 | Apply recycling systems, such as the use of solar energy, wind energy, green wind power, etc. | √ | [Liao et al. (2017) [5]](#_ENREF_5), [Loosemore et al. (2018) [6]](#_ENREF_6), [Wang et al. (2020) [18]](#_ENREF_18), [Maignan et al. (2000) [21]](#_ENREF_21), [Zhao et al. (2012) [28]](#_ENREF_28), [Zhao et al. (2016) [27]](#_ENREF_27), [Loosemore et al. (2018) [17]](#_ENREF_17), [Xie et al. (2020) [8]](#_ENREF_8), [Zhang et al. (2022) [9]](#_ENREF_9), [Bevan et al. (2015) [37]](#_ENREF_37), [Zeng et al. (2015) [32]](#_ENREF_32), [Lin et al. (2017) [7]](#_ENREF_7), [Loosemore et al. (2017) [38]](#_ENREF_38), [Zhang et al. (2019) [29]](#_ENREF_29), [Jones et al. (2006) [25]](#_ENREF_25), [Huang et al. (2012) [43]](#_ENREF_43), [Wuttke et al. (2014) [45]](#_ENREF_45), [Brown et al. (2009) [39]](#_ENREF_39), [Guo et al. (2021) [40]](#_ENREF_40), [Lu et al. (2016) [34]](#_ENREF_34) |
| E05 | Apply pollution emission control system, such as harmful gas, waste water, noise, dust, solid waste, etc. | √ | [Liao et al. (2017) [5]](#_ENREF_5), [Loosemore et al. (2018) [6]](#_ENREF_6), [Wang et al. (2020) [18]](#_ENREF_18), [Xie et al. (2020) [8]](#_ENREF_8), [Zhang et al. (2022) [9]](#_ENREF_9), [Zhao et al. (2012) [28]](#_ENREF_28), [Bevan et al. (2015) [37]](#_ENREF_37), [Zeng et al. (2015) [32]](#_ENREF_32), [Lin et al. (2017) [33]](#_ENREF_33), [Zhang et al. (2019) [29]](#_ENREF_29), [Jones et al. (2006) [25]](#_ENREF_25), [Huang et al. (2012) [43]](#_ENREF_43), [Brown et al. (2009) [39]](#_ENREF_39), [Guo et al. (2021) [40]](#_ENREF_40), [Lu et al. (2016) [34]](#_ENREF_34) |
| E06 | Use innovative technology and environmental protection technology | √ | [Liao et al. (2017) [5]](#_ENREF_5), [Loosemore et al. (2018) [6]](#_ENREF_6), [Wang et al. (2020) [18]](#_ENREF_18), [Zhao et al. (2016) [27]](#_ENREF_27), [Loosemore et al. (2018) [17]](#_ENREF_17), [Xie et al. (2020) [8]](#_ENREF_8), [Zhang et al. (2022) [9]](#_ENREF_9), [Zhao et al. (2012) [28]](#_ENREF_28), [Zeng et al. (2015) [32]](#_ENREF_32), [Loosemore et al. (2017) [38]](#_ENREF_38), [Jones et al. (2006) [25]](#_ENREF_25), [Huang et al. (2012) [43]](#_ENREF_43) |
| E07 | Implement emergency response mechanisms for environmental accidents | √ | [Jiang et al. (2016) [19]](#_ENREF_19), [Bevan et al. (2015) [37]](#_ENREF_37) |
| E08 | Pay attention to ecological restoration and governance, and ecosystem protection plans | √ | [Jiang et al. (2016) [19]](#_ENREF_19), [Zeng et al. (2015) [32]](#_ENREF_32), [Lin et al. (2017) [7]](#_ENREF_7), [Jones et al. (2006) [25]](#_ENREF_25), [Yam (2013) [30]](#_ENREF_30), [Brown et al. (2009) [39]](#_ENREF_39) |
| E09 | Promote/develop green office | √ | [Zhang et al. (2022) [9]](#_ENREF_9), Nguyen and Ha (2023) |
| E10 | Carry out green design and operations | √ | [Loosemore et al. (2018) [6]](#_ENREF_6), [Sabini et al. (2019) [14]](#_ENREF_14), [Wang et al. (2020) [18]](#_ENREF_18), [Zhao et al. (2016) [27]](#_ENREF_27), [Xie et al. (2020) [8]](#_ENREF_8), [Zhang et al. (2022) [9]](#_ENREF_9), [Wu et al. (2015) [11]](#_ENREF_11), [Zhao et al. (2012) [28]](#_ENREF_28), [Bevan et al. (2015) [37]](#_ENREF_37), [Zeng et al. (2015) [32]](#_ENREF_32), [Lin et al. (2017) [7]](#_ENREF_7), [Zhang et al. (2019) [29]](#_ENREF_29), [Yam (2013) [30]](#_ENREF_30), Nguyen and Ha (2023) |
| E11 | Support green and low-carbon business | √ | [Zhao et al. (2016) [27]](#_ENREF_27), [Lin et al. (2017) [33]](#_ENREF_33) |
| E12 | Carry out environmental protection public welfare activities | √ | [Zhao et al. (2016) [27]](#_ENREF_27) |
| E13 | Protect biodiversity | √ | [Loosemore et al. (2018) [6]](#_ENREF_6), [Martens et al. (2017) [35]](#_ENREF_35), [Loosemore et al. (2018) [17]](#_ENREF_17), [Zhang et al. (2022) [9]](#_ENREF_9), [Zeng et al. (2015) [32]](#_ENREF_32), [Zhang et al. (2019) [29]](#_ENREF_29), [Jones et al. (2006) [25]](#_ENREF_25), [Yam (2013) [30]](#_ENREF_30), [Ye et al. (2020) [46]](#_ENREF_46), [Brown et al. (2009) [39]](#_ENREF_39), [Lu et al. (2016) [34]](#_ENREF_34) |
| E14 | Implement land-use efficiency management mechanism | √ | [Zhao et al. (2012) [28]](#_ENREF_28), [Zhao et al. (2016) [27]](#_ENREF_27), [Loosemore et al. (2018) [17]](#_ENREF_17), [Xie et al. (2020) [8]](#_ENREF_8), [Zhang et al. (2022) [9]](#_ENREF_9), [Lin et al. (2017) [33]](#_ENREF_33), [Jones et al. (2006) [25]](#_ENREF_25) |

Notes: the CSR report tools include:

- Major international principles and codes such as: The Global Reporting Initiative (GRI 4.0), ISO 26000:2010, SA8000, the 2030 Agenda for Sustain, Guidelines for sustainability reporting in the oil and gas industry, Environmental, Social and Governance Reporting Guidelines (ESG).
- Chinese national and Stock Exchange requirement: Guidance on social responsibility reporting (GB/T36001-2015), Guidelines for Compiling Chinese Corporate Social Responsibility Reports (CASS-CSR4.0), the Guide on Social Responsibility for Chinese International Contractors issued by the Chinese International Contractors Association (CHINCA, 2012).

# References

1. Too E G, Weaver P. The management of project management: A conceptual framework for project governance. International Journal of Project Management. 2014; 32(8): 1382-1394.

2. Othman A, Abdellatif M. Partnership for integrating the corporate social responsibility of project stakeholders towards affordable housing development A South African perspective. Journal of Engineering Design and Technology. 2011; 9(3): 273-295.

3. Evangelinos K, Skouloudis A, Jones N, Isaac D, Sfakianaki E. Exploring the status of corporate social responsibility disclosure in the UK building and construction industry. International Journal of Global Environmental Issues. 2016; 15(4): 377-399.

4. Mayr S. Corporate social responsibility in SMEs: The case of an Austrian construction company. International Journal of Business Research. 2015; 15: 61-72.

5. Liao P C, Xia N N, Wu C L, Zhang X L, Yeh J L. Communicating the corporate social responsibility (CSR) of international contractors: Content analysis of CSR reporting. Journal of Cleaner Production. 2017; 156: 327-336.

6. Loosemore M, Lim B T H, Ling F Y Y, Zeng H Y. A comparison of corporate social responsibility practices in the Singapore, Australia and New Zealand construction industries. Journal of Cleaner Production. 2018; 190: 149-159.

7. Lin H, Zeng S, Ma H, Zeng R, Tam V W Y. An indicator system for evaluating megaproject social responsibility. International Journal of Project Management. 2017; 35(7): 1415-1426.

8. Xie L, Xu T, Le Y, Chen Q, Xia B, Skitmore M. Understanding the CSR awareness of large construction enterprises in China. Advances in Civil Engineering. 2020; 2020: 8866511.

9. Zhang Q, Oo B L, Lim B T-H. Key practices and impact factors of corporate social responsibility implementation: Evidence from construction firms. Engineering, Construction and Architectural Management. 2022; ahead-of-print(ahead-of-print).

10. Zhang Q, Lan Oo B, Lim Benson Teck H. Mapping Perceptions and Implementation of Corporate Social Responsibility for Construction Firms via Importance–Performance Analysis: Paths of Improvement. Journal of Management in Engineering. 2021; 37(6): 04021061.

11. Wu C L, Fang D P, Liao P C, Xue J W, Li Y, Wang T. Perception of corporate social responsibility: the case of Chinese international contractors. Journal of Cleaner Production. 2015; 107: 185-194.

12. Petrovic‐Lazarevic S. The development of corporate social responsibility in the Australian construction industry. Construction Management and Economics. 2008; 26(2): 93-101.

13. Liao P C, Xue J, Liu B, Fang D. Selection of the approach for producing a weighting scheme for the CSR evaluation framework. KSCE Journal of Civil Engineering. 2015; 19(6): 1549-1559.

14. Sabini L, Muzio D, Alderman N. 25 years of 'sustainable projects'. What we know and what the literature says. International Journal of Project Management. 2019; 37(6): 820-838.

15. Martens M L, Carvalho M M. The challenge of introducing sustainability into project management function: multiple-case studies. Journal of Cleaner Production. 2016; 117: 29-40.

16. Silvius G. Sustainability as a competence of Project Managers. PM World Journal. 2016; 5: 1-13.

17. Loosemore M, Lim B T H. Mapping corporate social responsibility strategies in the construction and engineering industry. Construction Management and Economics. 2018; 36(2): 67-82.

18. Wang L, Zhang P, Ma L, Cong X, Skibniewski M J. Developing a corporate social responsibility framework for sustainable construction using partial least squares structural equation modeling. Technological and Economic Development of Economy. 2020; 26(1): 186-212.

19. Jiang W, Wong J K W. Key activity areas of corporate social responsibility (CSR) in the construction industry: a study of China. Journal of Cleaner Production. 2016; 113: 850-860.

20. Daily C, Dalton D, Cannella A. Introduction to special topic forum corporate governance: Decades of dialogue and data. The Academy of Management Review. 2003; 28(3): 371-382.

21. Maignan I, Ferrell O C. Measuring Corporate Citizenship in Two Countries: The Case of the United States and France. Journal of Business Ethics. 2000; 23(3): 283-297.

22. Gorden W I, Anderson C M, Bruning S D. Employee perceptions of corporate partnership: An affective-moral quid pro quo. Employee Responsibilities and Rights Journal. 1992; 5(1): 75-85.

23. Manowong E, Ogunlana S O. Public hearings in Thailand's infrastructure projects: effective participations? Engineering Construction and Architectural Management. 2006; 13(4): 343-363.

24. Oppong G D, Chan A P C, Dansoh A. A review of stakeholder management performance attributes in construction projects. International Journal of Project Management. 2017; 35(6): 1037-1051.

25. Jones P, Comfort D, Hillier D. Corporate social responsibility and the UK construction industry. Journal of Corporate Real Estate. 2006; 8(3): 134-150.

26. Turker D. Measuring corporate social responsibility: A scale development study. Journal of Business Ethics. 2009; 85(4): 411-427.

27. Zhao Z-Y, Zhao X-J, Zuo J, Zillante G. Corporate social responsibility for construction contractors: a China study. Journal of Engineering, Design and Technology. 2016; 14(3): 614-640.

28. Zhao Z Y, Zhao X J, Davidson K, Zuo J. A corporate social responsibility indicator system for construction enterprises. Journal of Cleaner Production. 2012; 29-30: 277-289.

29. Zhang Q, Oo B L, Lim B T H. Drivers, motivations, and barriers to the implementation of corporate social responsibility practices by construction enterprise: A review. Journal of Cleaner Production. 2019; 210: 563-584.

30. Yam S. The practice of corporate social responsibility by Malaysian developers. Property Management. 2013; 31(1): 76-91.

31. Nguyen M V. Investigating the relationship between corporate social responsibility implementation and contractor competitiveness. Engineering, Construction and Architectural Management. 2023; ahead-of-print(ahead-of-print).

32. Zeng S X, Ma H Y, Lin H, Zeng R C, Tam V W Y. Social responsibility of major infrastructure projects in China. International Journal of Project Management. 2015; 33(3): 537-548.

33. Lin X, Ho C M F, Shen G Q P. Who should take the responsibility? Stakeholders' power over social responsibility issues in construction projects. Journal of Cleaner Production. 2017; 154: 318-329.

34. Lu W, Ye M, Flanagan R, Ye K. Corporate Social Responsibility Disclosures in International Construction Business: Trends and Prospects. Journal of Construction Engineering and Management. 2016; 142(1): 04015053.

35. Martens M L, Carvalho M M. Key factors of sustainability in project management context: A survey exploring the project managers' perspective. International Journal of Project Management. 2017; 35(6): 1084-1102.

36. Darko A, Chan A P C. Critical analysis of green building research trend in construction journals. Habitat International. 2016; 57: 53-63.

37. Bevan E a M, Yung P. Implementation of corporate social responsibility in Australian construction SMEs. Engineering, Construction and Architectural Management. 2015; 22(3): 295-311.

38. Loosemore M, Lim B T H. Linking corporate social responsibility and organizational performance in the construction industry. Construction Management and Economics. 2017; 35(3): 90-105.

39. Brown J, Parry T, Moon J. Corporate responsibility reporting in UK construction. Proceedings of the Institution of Civil Engineers - Engineering Sustainability. 2009; 162(4): 193-205.

40. Guo H, Lu W. The inverse U-shaped relationship between corporate social responsibility and competitiveness: Evidence from Chinese international construction companies. Journal of Cleaner Production. 2021; 295: 126374.

41. Gao-Zeller X J, Li X D, Yang F, Zhu W N. Driving Mechanism of CSR Strategy in Chinese Construction Companies based on Neo-Institutional Theory. KSCE JOURNAL OF CIVIL ENGINEERING. 2019; 23(5): 1939-1951.

42. Li X, Gao-Zeller X, Rizzuto T E, Yang F. Institutional pressures on corporate social responsibility strategy in construction corporations: The role of internal motivations. Corporate Social Responsibility and Environmental Management. 2019; 26(4): 721-740.

43. Huang C-F, Lien H-C. An empirical analysis of the influences of corporate social responsibility on organizational performance of Taiwan’s construction industry: using corporate image as a mediator. Construction Management and Economics. 2012; 30(4): 263-275.

44. Velychko V, Prunenko D, Grytskov E. Corporate Social Responsibility in The System of Interaction Between Stakeholders of Construction Enterprises. Baltic Journal of Economic Studies. 2020; 6(5): 64-72.

45. Wuttke M, Vilks A. Poverty alleviation through CSR in the Indian construction industry. Journal of Management Development. 2014; 33(2): 119-130.

46. Ye M, Lu W, Flanagan R, Chau K W. Corporate social responsibility 'glocalisation': Evidence from the international construction business. Corporate Social Responsibility & Environmental Management. 2020; 27(2): 655-669.

47. Maignan I, Hillebrand B, Mcalister D. Managing Socially-Responsible Buying:: How to Integrate Non-economic Criteria into the Purchasing Process. European Management Journal. 2002; 20(6): 641-648.

48. Li W, Zhang R. Corporate Social Responsibility, Ownership Structure, and Political Interference: Evidence from China. Journal of Business Ethics. 2010; 96(4): 631-645.

49. Leigh A, Neill C. Can national infrastructure spending reduce local unemployment? Evidence from an Australian roads program. Economics Letters. 2012; 113(2): 150-153.

50. Barthorpe S. Implementing corporate social responsibility in the UK construction industry. Property Management. 2010; 28(1): 4-17.

51. Alzahrani J I, Emsley M W. The impact of contractors’ attributes on construction project success: A post construction evaluation. International Journal of Project Management. 2013; 31(2): 313-322.

52. Sonja P-L. The development of corporate social responsibility in the Australian construction industry. Construction Management and Economics. 2008; 26(2): 93-101.
