# Supplementary material for: Mapping stakeholders’ relationships management in fulfilling corporate social responsibility: A study of China’s construction industry
Source: PLoS One. 2024 Jan 5;19(1):e0294588. doi: 10.1371/journal.pone.0294588 (PMC10769051; doi:10.1371/journal.pone.0294588)
Supplement: S3 Table — (DOCX) [file pone.0294588.s004.docx]

**S-Table 3. ENR’s 2022 Top 10 Chinese International Contractors.**

| **2022 ENR Ranking** | **Name** |
| --- | --- |
| 3 | CHINA COMMUNICATIONS CONSTRUCTION GROUP LTD. |
| 6 | POWER CONSTRUCTION CORP. OF CHINA |
| 7 | CHINA STATE CONSTRUCTION ENGINEERING CORP. LTD. |
| 10 | CHINA RAILWAY CONSTRUCTION CORP. LTD. |
| 11 | CHINA RAILWAY GROUP LTD. |
| 17 | CHINA ENERGY ENGINEERING CORP. LTD. |
| 20 | CHINA NATIONAL CHEMICAL ENG'G GROUP CORP. LTD. |
| 28 | CHINA NATIONAL MACHINERY INDUSTRY CORP. |
| 30 | CHINA PETROLEUM ENGINEERING CO. |
| 40 | SHANGHAI ELECTRIC GROUP COMPANY LIMITED |

Note: please see the website:

<https://www.enr.com/toplists/2022-Top-250-International-Contractors-Preview>
