# Supplementary material for: Mapping stakeholders’ relationships management in fulfilling corporate social responsibility: A study of China’s construction industry
Source: PLoS One. 2024 Jan 5;19(1):e0294588. doi: 10.1371/journal.pone.0294588 (PMC10769051; doi:10.1371/journal.pone.0294588)
Supplement: S4 Table — (DOCX) [file pone.0294588.s005.docx]

# S-Table 4. The stakeholder - CSR matrix framework (S-CSR-M).

| CSR issues | Codes | CSR Activities | Government | Shareholders | Employees | Suppliers | End users | Community group | Environment and resources agencies | Partners | Supervisor | Competitors | Union | Minority | NGOs and Media |
| --- | --- | --- | --- | --- | --- | --- | --- | --- | --- | --- | --- | --- | --- | --- | --- |
| Responsible Management (R) | R01 | Develop CSR plans | √ |  |  |  |  |  |  |  |  |  |  |  |  |
|  | R02 | Promote the integration of CSR into corporate strategies and routine | √ | √ |  |  |  |  |  |  |  |  |  |  |  |
|  | R03 | Guide managers to participate in CSR |  | √ |  |  |  |  |  |  |  |  |  |  |  |
|  | R04 | Establish CSR leadership bureau and mechanisms | √ |  |  |  |  |  |  |  |  |  |  |  |  |
|  | R05 | Make CSR management systems and set up departments | √ |  |  |  |  |  |  |  |  |  |  |  |  |
|  | R06 | Develop/organize CSR training | √ |  |  |  |  |  |  |  |  |  |  |  |  |
|  | R07 | Participate in the making of CSR standards | √ |  |  |  |  |  |  |  |  |  |  |  |  |
|  | R08 | Establish communication mechanisms | √ |  |  |  |  |  |  |  |  |  |  |  |  |
|  | R09 | Join CSR organization/convention | √ |  |  |  |  |  |  |  |  | √ |  |  |  |
|  | R10 | CSR crisis management | √ | √ |  |  |  |  |  |  |  |  |  |  |  |
|  | R11 | CSR third-party assessment | √ | √ |  |  |  |  |  |  |  |  |  |  | √ |
| Corporate Governance (G) | G01 | Improve corporate governance structure | √ | √ |  |  |  |  |  |  |  |  |  |  |  |
|  | G02 | Invest resources to innovate the corporate management | √ | √ |  |  |  |  |  |  |  | √ |  |  |  |
|  | G03 | legal management | √ | √ |  |  | √ |  |  | √ | √ | √ | √ |  | √ |
|  | G04 | Maintain investor relations and protect shareholder rights | √ | √ |  |  |  |  |  |  |  |  |  |  |  |
|  | G05 | Disclosure information to improve transparency | √ | √ |  |  | √ |  |  | √ | √ | √ |  |  | √ |
|  | G06 | Prohibition of bribery and corruption | √ |  |  | √ |  |  |  | √ | √ |  |  |  | √ |
|  | G07 | Establish operating mechanism to improve the profit | √ | √ |  |  |  |  |  |  |  |  |  |  |  |
|  | G08 | Promote corporate brand |  | √ |  |  |  |  |  |  |  | √ |  |  |  |
| Safe Construction and Quality (Q) | Q01 | Acquire quality management certification | √ |  |  |  | √ |  |  | √ |  |  |  |  |  |
|  | Q02 | Optimize product/service quality management system | √ |  |  |  | √ |  |  | √ |  |  |  |  |  |
|  | Q03 | Establish R&D and innovation system | √ |  |  |  | √ |  |  | √ |  | √ |  |  |  |
|  | Q04 | Develop customer-oriented product |  |  |  |  | √ |  |  | √ |  |  |  |  |  |
|  | Q05 | Product warnings |  |  |  |  | √ |  |  | √ |  |  |  |  | √ |
|  | Q06 | Ensure fair product/service transaction | √ |  |  |  | √ |  |  | √ |  |  |  |  |  |
|  | Q07 | Advocate sustainable consumption |  |  |  |  | √ |  |  | √ |  |  |  |  |  |
|  | Q08 | Protect consumer privacy | √ |  |  |  | √ |  |  | √ |  |  |  |  | √ |
|  | Q09 | Establish / improve after-sales service system | √ |  |  |  | √ |  |  | √ |  |  |  |  | √ |
|  | Q10 | Implement/optimize quality training |  |  |  |  |  |  |  | √ |  |  |  |  |  |
|  | Q11 | Employee safety education and training | √ |  | √ |  |  |  |  |  |  |  |  |  |  |
|  | Q12 | Implement/optimize safety production management system |  |  |  |  |  |  |  | √ | √ |  |  |  |  |
|  | Q13 | Application/optimization of security management prevention mechanism |  |  |  |  |  |  |  | √ | √ |  |  |  |  |
|  | Q14 | Implement safety responsibility management | √ |  |  |  |  |  |  | √ | √ |  |  |  |  |
|  | Q15 | Establish regular communication mechanisms with customers | √ |  |  |  | √ |  |  |  |  |  |  |  | √ |
| Good Partnership (P) | P01 | Implement/apply supply chain management mechanism | √ |  |  | √ |  |  |  |  |  |  |  |  |  |
|  | P02 | Carry out supplier CSR training |  |  |  | √ |  |  |  |  |  |  |  |  |  |
|  | P03 | Establish a partner communication mechanism to improve cooperation |  |  |  | √ |  |  |  | √ | √ |  |  |  | √ |
|  | P04 | Protect intellectual property | √ |  |  |  |  |  |  | √ | √ |  |  |  | √ |
|  | P05 | Communication within the industry to promote industry development | √ |  |  |  |  |  |  | √ | √ | √ |  |  | √ |
|  | P06 | Alliance with other companies, institutions, groups, etc. |  |  |  |  |  |  |  | √ | √ | √ |  |  | √ |
|  | P07 | Building a green supply chain |  |  |  | √ |  |  | √ |  |  |  |  |  |  |
| Workers Interest (W) | W01 | Implement employee career development management system |  |  | √ |  |  |  |  |  |  |  | √ |  |  |
|  | W02 | Establish an employee promotion mechanism |  |  | √ |  |  |  |  |  |  |  | √ |  |  |
|  | W03 | Implement employer/employee relationship management |  |  | √ |  |  |  |  |  |  |  | √ |  |  |
|  | W04 | Implement effective denial, report and complaint mechanisms through the labor unions | √ |  | √ |  |  |  |  |  |  |  | √ |  |  |
|  | W05 | Implement employee and family care plan |  |  | √ |  |  |  |  |  |  |  | √ |  |  |
|  | W06 | Protect employees’ occupational health and safety | √ |  | √ |  |  |  |  |  |  |  | √ |  |  |
|  | W07 | Protect the rights of migrant workers | √ |  | √ |  |  |  |  |  |  |  | √ |  |  |
|  | W08 | Advocate the multicultural and individual development of employees |  |  | √ |  |  |  |  |  |  |  | √ |  |  |
|  | W09 | Protect employee privacy | √ |  | √ |  |  |  |  |  |  |  | √ |  |  |
|  | W10 | Guarantee the working environment and working conditions of employees |  |  | √ |  |  |  |  |  |  |  | √ |  |  |
|  | W11 | Sign labor contracts in accordance with the law to combat forced labor | √ |  | √ |  |  |  |  |  |  |  | √ |  | √ |
|  | W12 | Prohibition of harassment and abuse of employees |  |  | √ |  |  |  |  |  |  |  | √ |  | √ |
|  | W13 | Equal employment | √ |  | √ |  |  |  |  |  |  |  | √ |  |  |
|  | W14 | Effective emergency management procedures and safety monitoring mechanisms |  |  | √ |  |  |  |  |  |  |  |  |  |  |
| Well-being of Local Community (C) | C01 | Assess public and community needs |  |  |  |  |  | √ |  |  |  |  |  |  |  |
|  | C02 | Establish effective communication channels and participation mechanisms |  |  |  |  |  | √ |  |  |  |  |  |  |  |
|  | C03 | Handle community complaints promptly |  |  |  |  |  | √ |  |  |  |  |  |  | √ |
|  | C04 | Organize community vocational skills training programs and knowledge popularization activities |  |  |  |  |  | √ |  |  |  |  |  |  |  |
|  | C05 | Priority purchase of local products and services |  |  |  |  |  | √ |  |  |  |  |  |  |  |
|  | C06 | Protect local culture | √ |  |  |  |  | √ |  |  |  |  |  |  |  |
|  | C07 | Disaster prevention/relief activities | √ |  |  |  |  | √ |  |  |  |  |  |  |  |
|  | C08 | Support the development of infrastructure and public services in local communities | √ |  |  |  |  | √ |  |  |  |  |  |  |  |
|  | C09 | Pay attention to the rights and interests of disadvantaged group | √ |  |  |  |  | √ |  |  |  |  |  | √ |  |
|  | C10 | Protect the rights of local ethnic minorities | √ |  |  |  |  | √ |  |  |  |  |  | √ |  |
|  | C11 | Alleviate community poverty | √ |  |  |  |  | √ |  |  |  |  |  | √ | √ |
|  | C12 | Prioritize local employees |  |  | √ |  |  | √ |  |  |  |  |  | √ |  |
|  | C13 | Establish a charity fund or foundation |  |  |  |  |  | √ |  |  |  |  |  | √ | √ |
|  | C14 | Formulate policies and measures to support volunteer activities |  |  |  |  |  | √ |  |  |  |  |  | √ | √ |
| Environment Preservation (E) | E01 | Establish environmental management system | √ |  |  |  |  |  | √ |  |  |  |  |  | √ |
|  | E02 | Implement/optimize staff’s environmental training |  |  | √ |  |  |  | √ |  |  |  |  |  |  |
|  | E03 | Apply/optimize the environmental impact assessment before construction |  |  |  |  | √ |  | √ | √ | √ |  |  |  |  |
|  | E04 | Apply recycling systems |  |  |  |  |  |  | √ |  |  |  |  |  | √ |
|  | E05 | Apply pollution emission control system | √ |  |  |  |  |  | √ |  | √ |  |  |  | √ |
|  | E06 | Use innovative technology and environmental protection technology |  |  |  |  |  |  | √ |  |  |  |  |  | √ |
|  | E07 | Implement emergency response mechanisms for environmental accidents |  |  |  |  |  | √ | √ |  |  |  |  |  | √ |
|  | E08 | Pay attention to ecological restoration | √ |  |  |  |  | √ | √ |  |  |  |  |  | √ |
|  | E09 | Promote/develop green office |  |  |  |  |  |  | √ |  |  |  |  |  |  |
|  | E10 | Carry out green design and construction |  |  |  |  |  | √ | √ | √ | √ |  |  |  |  |
|  | E11 | Support green and low-carbon business |  |  |  |  |  | √ | √ |  |  |  |  |  |  |
|  | E12 | Carry out environmental protection public welfare activities |  |  |  |  |  |  | √ |  |  |  |  |  | √ |
|  | E13 | Protect biodiversity | √ |  |  |  |  | √ | √ |  | √ |  |  | √ | √ |
|  | E14 | Implement land use efficiency management mechanism | √ |  |  |  |  | √ | √ |  |  |  |  |  | √ |
|  |  |  |  |  |  |  |  |  |  |  |  |  |  |  |  |

Note: According to Zhao et al. (2012) [1], Zeng et al. (2015) [2], Wu et al. (2015) [3], Zhao et al. (2016) [4], Lin et al. (2017) [5], Velychko et al. (2020) [6], and the reports of “ENR’s 2022 Top 10 Chinese International Contractors” in 2010, 2015, 2020.

# References

1. Zhao Z Y, Zhao X J, Davidson K, Zuo J. A corporate social responsibility indicator system for construction enterprises. Journal of Cleaner Production. 2012; 29-30: 277-289.

2. Zeng S X, Ma H Y, Lin H, Zeng R C, Tam V W Y. Social responsibility of major infrastructure projects in China. International Journal of Project Management. 2015; 33(3): 537-548.

3. Wu C L, Fang D P, Liao P C, Xue J W, Li Y, Wang T. Perception of corporate social responsibility: the case of Chinese international contractors. Journal of Cleaner Production. 2015; 107: 185-194.

4. Zhao Z-Y, Zhao X-J, Zuo J, Zillante G. Corporate social responsibility for construction contractors: a China study. Journal of Engineering, Design and Technology. 2016; 14(3): 614-640.

5. Lin H, Zeng S, Ma H, Zeng R, Tam V W Y. An indicator system for evaluating megaproject social responsibility. International Journal of Project Management. 2017; 35(7): 1415-1426.

6. Velychko V, Prunenko D, Grytskov E. Corporate Social Responsibility in The System of Interaction Between Stakeholders of Construction Enterprises. Baltic Journal of Economic Studies. 2020; 6(5): 64-72.
