# Supplementary material for: Mapping stakeholders’ relationships management in fulfilling corporate social responsibility: A study of China’s construction industry
Source: PLoS One. 2024 Jan 5;19(1):e0294588. doi: 10.1371/journal.pone.0294588 (PMC10769051; doi:10.1371/journal.pone.0294588)
Supplement: Attachment — Submitted filename: Review Report_PONE-D-23-23102.docx [file pone.0294588.s006.docx]

**REVIEW REPORT**

**PONE-D-23-23102**

**Mapping stakeholders’ relationships management in fulfilling corporate social responsibility: A study of China’s construction industry**

I welcome the authors’ efforts to investigate the link between stakeholders’ relationships management and corporate social responsibility. I think the paper is addressing an important issue. However, as you might have expected, I do have some concerns and I would like to explain my concerns and suggestions below, in hopes of helping the authors improve the paper.

1. In my view, authors can also use the lens of legitimacy theory to further support their argument regarding CSR implementation in response of stakeholders’ management/relationship in the context of construction industry. For legitimacy theory, authors can refer the following studies, among others.

Di Vaio, A., Varriale, L., Di Gregorio, A., & Adomako, S. (2022). Corporate social performance and non‐financial reporting in the cruise industry: Paving the way towards UN Agenda 2030. Corporate Social Responsibility and Environmental Management, 29(6), 1931-1953.

Velte, P. (2022). Meta-analyses on corporate social responsibility (CSR): a literature review. Management Review Quarterly, 72(3), 627-675.

2. In the literature review section, I would suggest authors to add hypothesis for each link, for instance, regarding stakeholder-based view on CSR and stakeholder relationship management.

3. In the abstract, authors mentioned that “the results show that the SCSR-2M can be composed of three key stakeholders, namely government, partners, NGOs and media.” I think these are four stakeholders not three or authors might consider both NGOs and media as one stakeholder. Confirm the same in rest of the manuscript.

4. Finally, the paper should go through English proof editing. The paper is not very clear to the reader.
