# Supplementary material for: Mapping stakeholders’ relationships management in fulfilling corporate social responsibility: A study of China’s construction industry
Source: PLoS One. 2024 Jan 5;19(1):e0294588. doi: 10.1371/journal.pone.0294588 (PMC10769051; doi:10.1371/journal.pone.0294588)
Supplement: Attachment — Submitted filename: Response to Reviewers.docx [file pone.0294588.s007.docx]

**Rebuttal Letter: Response to Editor and Reviewers’ Comments**

**Manuscript title:** Mapping stakeholders’ relationships management in fulfilling corporate social responsibility: A study of China’s construction industry

**Manuscript ID: PONE-D-23-23102**

Dear Editor,

Thank you for considering our research manuscript and giving us the opportunity to revise the paper for potential publication in ***PLOS ONE***. Special thanks go to the editor and reviewers for their constructive comments. These comments are very helpful for us to improve the paper. We have addressed all of the reviewers’ comments carefully and have made major revision to the paper.

Based on the reviewers’ comments, we have made the following major revisions to the manuscript, including: 1) Introducing the basic situation of China’s construction industry as well as the characteristics of CSR. 2) Re-writing Chapter [2. Literature Review], including introducing three hypotheses. 3) Reorganize the methodological steps of this study, redraw the flowchart and adjust the relevant parts. 4) Combining Chapter [5. Results and Discussion] with Chapter [2. Literature Review] to further analysis and explain the results of this study. 5) Adjusting the paragraphs of the original manuscript. 6) Proofread the manuscript.

We would like to resubmit the paper to the Journal for peer review. We hope that the revised manuscript meets the requirements.

Many thanks and best regards.

Yours sincerely,

Kunhui Ye

-------------------------------------------------------------------------------------------------------
Please find our responses to reviewers’ comments as follows.

| **Editor’s Comments** | **Authors’ Responses** |
| --- | --- |
| **Reviewer #1** | |
| **Reviewer’s Comments** | **Authors’ Responses** |
| The reviewer’s main concern is about the **literature review**. | We are grateful for the Reviewer #1’s detailed comments and revision suggestions on this manuscript.  **Please see *[2 Literature Review]*.** |
| 1.1 Ideas are jumbled together in a way that makes it difficult for readers to closely follow along. The literature review, for instance, cites numerous studies, but it is unclear how (many of) the cited studies relate to the present research (in a way that advances arguments or builds theoretical predictions). Despite the coverage of the literature review, which claims are relevant for the present research is unclear. It is my belief that the front-end of the manuscript would need significant re-writing to improve concision, clarity, and structure. | Thanks for your comments. Firstly, the basics of CSR activities in the construction industry are presented and “*Hypothesis 1: Companies prioritize the implementing of key CSR activities and leverage their associated relationships in the fulfillment process*” is proposed. Then the stakeholder-based view in CSR is introduced and “*Hypothesis 2: Companies prioritize meeting the needs of key stakeholders*” is proposed. Finally, on the basis of the above two parts, *“Hypothesis 3: There are associated relationships between stakeholders”* of this study is proposed by combining the legitimacy theory, the resource-based perspective (which implies the potential associated relationships between stakeholders in CSR).  Based on the above, we have rewritten the part of **[2. Literature Review]**.  **Please see *[2 Literature Review]*.** |
| 1.2 Which specific studies motivate an investigation into China's construction industry? | Thank you for the comments. We add the explanation of choosing China’s construction industry as the research object. The basic situation of China’s construction industry, especially in terms of resources consumption, the basic situation and characteristics of CSR development in China’s construction industry, as well as comparisons with other countries are presented.  **Please see lines 45-59:**  *“According to the National Bureau of Statistics of China (2022), China’s construction industry has an output value of RMB 311,979.84 billion and 51,480,200 employees. However, the construction industry in China consumed 45.5% of energy and generated 50.9% of carbon emissions in 2020 [**11**], suggesting that the industry has become a major pollutant source. The increasing problem of unsustainability in the construction industry draws considerable attention from researchers to explore more solutions. For instance,* *Wu et al. (2015) [12]* *found it worthy of highlighting the importance of CSR in the management of construction quality and safety;* *Chang et al. (2018) [13]* *argued that quality management and customer services should be connected to the fulfillment of CSR. While practitioners’ CSR awareness stays low [**14**,* *15**], progress has been made in China to embrace communication, biodiversity, ecological conservation, and employee education [**16**]. Unlike developed countries where climate change, ethics, and sustainable technologies are highly recognized, China’s construction business is beginning to introduce stakeholder management into CSR strategies to achieve a more balanced approach with policy.”* |
| 1.3 How do CSR initiatives concretely differ based on industries? What attributes lead to these differences? | Thank you for the questions. We have added the concerns to the study to point out the attributes of the construction industry and the differences and characteristics that come about because of this attribute.  **Please see lines 36-40:**  *“CSR research in construction has gained prominence as a key agenda, possibly due to the two-sided uniqueness of construction business with its high positive and negative impacts [**7**]. Construction activities consume a large amount of resource, produce construction wastes, pollutants, and greenhouse gas emissions, and causes multidimensional impacts on the public and physical environment [**1**,* *8**,* *9**].”*  **And lines 135-146:**  *“CSR in construction differs from those in other sectors due to the need to address various paradoxes [**29**], focusing on maintaining responsible aspects of construction activities while eliminating irresponsible elements [**4**]. On the one hand, the construction industry provides considerable jobs and makes it socially responsible to support our social and economic activities [**30**]. On the other hand, construction activities are often criticized for their irresponsible attributes owing to the generation of considerable negative impacts on the natural environment and poor occupational health and safety records [**31**]. For example, CSR in construction companies has been shown to improve corporate competitiveness, financial performance, image and business reputation [**14**]; as well as contribute directly or indirectly to the achievement of some sustainable development goals (SDGs), such as the SDGs for food, health, education, women, water and energy [**4**].”* |
| 1.4 What do we already know about CSR in the construction industry? | Thanks for your comment. We further describe our understanding of CSR in the construction industry in the **[1. Introduction]** and **[2. literature review]**. This includes, the characteristics of CSR in the construction industry, the development of the literature, and the specifics of CSR activities in the construction industry.  **Please see lines 36-40:**  *“CSR research in construction has gained prominence as a key agenda, possibly due to the two-sided uniqueness of construction business with its high positive and negative impacts [**7**]. Construction activities consume a large amount of resource, produce construction wastes, pollutants, and greenhouse gas emissions, and causes multidimensional impacts on the public and physical environment [**1**,* *8**,* *9**].”*  **Please see *[2.1 CSR activities in construction],*** which introduces the current situation and development of CSR in the construction industry.  **Please see S1-2 Tables**, which summarizes and describes the specifics of CSR activities in the construction industry based on a literature search. |
| 1.5 How does the construction industry in China differ from the same industry in different countries? Might these structural differences lead to differences in CSR activities? | Thanks to the reviewer for the suggestions. We have added the characteristics of China’s construction industry and its CSR, as well as the differences from other countries in **[1. Introduction]**. This difference has also led Chinese construction CSR to place more emphasis on government-related CSR activities, as discussed in **[5.2 Key stakeholder involved in construction]**.  **Please see lines 45-59:**  *“According to the National Bureau of Statistics of China (2022), China’s construction industry has an output value of RMB 311,979.84 billion and 51,480,200 employees. However, the construction industry in China consumed 45.5% of energy and generated 50.9% of carbon emissions in 2020 [**11**], suggesting that the industry has become a major pollutant source. The increasing problem of unsustainability in the construction industry draws considerable attention from researchers to explore more solutions. For instance,* *Wu et al. (2015) [12]* *found it worthy of highlighting the importance of CSR in the management of construction quality and safety;* *Chang et al. (2018) [13]* *argued that quality management and customer services should be connected to the fulfillment of CSR. While practitioners’ CSR awareness stays low [**14**,* *15**], progress has been made in China to embrace communication, biodiversity, ecological conservation, and employee education [**16**]. Unlike developed countries where climate change, ethics, and sustainable technologies are highly recognized, China’s construction business is beginning to introduce stakeholder management into CSR strategies to achieve a more balanced approach with policy.”*  **And lines 473-481:**  *“While governments and construction companies may not have a direct contractual relationship, governments exert significant influence through laws and regulations, and companies are encouraged to utilize CSR to address social and environmental issues [**18**,* *80**]. Compared with other countries, Chinese construction companies have a unique governance model in which the Chinese government has greater control over large, state-owned construction companies. The management of these companies is appointed by the government and has major decision-making power in the company. Therefore, the influence of government on CSR has been highlighted.”* |
| 1.6 What justifies the methodological approach used in the current research? And does this approach differ from other methods used in CSR research? And why is the approach preferable to other potentially viable methods? | Thanks for the reviewer’s questions. This study uses a quantitative research method, which is divided into the following steps:  ***Step 1:*** Explore the associations relationships of CSR activities, including:  1) Constructing a framework of CSR activities in the construction industry through existing literature;  2) Collecting CSR reports from listed construction companies in China;  3) Mining the text of the reports collected in 2) for CSR activities in 1);  4) Employ association analysis of CSR activities;  ***Step 2:*** Establish a mapping matrix of CSR activities and stakeholders in the construction industry based on the literature and discover key stakeholders.  ***Step 3:*** Conduct association analysis of stakeholders and identify stakeholder types and positions.  We reorganized the methodological steps of this study and redrew the flowcharts.  **Please see lines 203-211 and Figure 1:**  *“Three steps are adopted to actualize the research objectives. First, we conduct a systematic literature review to identify the CSR activities and use text mining of the collected reports; then employ an association analysis of CSR activities. Second, we segment stakeholders corresponding to each CSR activity based on samples from the Engineering News Record (ENR) Chinese top 5 and related literature to establish a stakeholder - CSR matrix framework (S-CSR-M). Third, the associated CSR activities obtained in the first step are mapped to stakeholders through S-CSR-M; then the stakeholder relationships are obtained through association mining. The research methodology is outlined in Fig 1.”*    **Fig 1. The research ﬂow chart.**  This study uses association rule mining to explore the potential relationships of stakeholders in the CSR activities. This approach is different from other methods used in CSR research, such as the common social network analysis. A social network is defined as a specific set of linkages among a defined set of persons (Mitchell, [1969](https://www-tandfonline-com.libproxy1.nus.edu.sg/doi/full/10.1080/01446193.2010.521759)), which interprets the project environment as a system connected by various relationships that can be used to map the interrelationships between stakeholders and the social behavior of the people involved (Yang et al., 2011). Mok et al. (2015) also pointed out that the social network approach can understand the stakeholder environment by considering the structural characteristics of the stakeholder network and the interactions among multiple stakeholders. Therefore, social network analysis is often used for stakeholder relationship analysis.  Association rule mining (ARM) is a widely used data mining method that can identify hidden information in data to discover close relationships between items in a large amount of data (Agrawal, Imieliński and Swami, 1993), and discover knowledge discovery data (KDD) to explain specific issues (Le and Vo, 2016), is increasingly important for supporting decision making processes of companies and organizations. An association rule quantitatively characterizes how the presence of a set of items in the records of a database indicate the presence of some other distinct set of elements in the same records (Agrawal, Imieliński and Swami, 1993). Thus, the aim of association rules is to identify trends that can be used to understand and explore patterns of behavior in data. For example, if we consider supermarket sales data, we may find that 80% of the customers who buy product Q also buy product W, and thus 80% corresponds to the reliability of this rule (Domingues and Rezende, 2005). The application of ARM in management science and engineering is increasing gradually. For example, Xu et al. (2014) studied its application in tunnel maintenance, Li et al. (2019) used this method to discover the relationship between industrialization level and air quality, Xu and Luo (2021) applied it to air traffic controller unsafe behavior risk prediction and early warning, and Abdirad and Mathur (2021) used this method to BIM content management and delivery, etc.  **References:**  [1] Mitchell R K, Agle B. Toward a Theory of Stakeholder Identification and Salience: Defining the Principle of who and What Really Counts. Academy of Management Review. 1997; 22(4): 853-886.  [2] Yang J, Shen G Q, Bourne L, Ho C M F, Xue X. A typology of operational approaches for stakeholder analysis and engagement. Construction Management and Economics. 2011; 29(2): 145-162.  [3] Mok K Y, Shen G Q, Yang J. Stakeholder management studies in mega construction projects: A review and future directions. International Journal of Project Management. 2015; 33(2): 446-457.  [4] Le T, Vo B. The lattice-based approaches for mining association rules: a review. Wiley Interdisciplinary Reviews Data Mining & Knowledge Discovery. 2016; 6(4): 140-151.  [5] Domingues, M A, Rezende, S O. Post-processing of Association Rules using Taxonomies. In: Proceedings of the 12th Portuguese Conference on Artifcial Intelligence. 2005:192-197.  [6] Junxue Z, Weixiang X, Yinhong F. Association Rules Algorithm and Its Application in the Maintenance of the Tunnel. International Journal of Simulation Modelling. 2014; 13: 458-471.  [7] Li T, Li Y, An D, Han Y, Xu S, Lu Z, Crittenden J. Mining of the association rules between industrialization level and air quality to inform high-quality development in China. Journal of Environmental Management. 2019; 246(564-574).  [8] Xu R, Luo F. Risk prediction and early warning for air traffic controllers’ unsafe acts using association rule mining and random forest. Safety Science. 2021; 135: 105125.  The choice of method for this study was determined by the meaning of the data. ARM can mine more data information from CSR behaviors, which is more suitable for the purpose of this research.  **Please see lines 269-274:**  *“Social network analysis (SNA) is also often used to map the interrelationships between stakeholders and social behaviors (e.g. [**24**], [**67**]). However, SNA may have limitations in boundary, bias and uncertainty [**24**]. ARM is increasingly applied in construction management research (e.g. [**68-70**]). Appling ARM to the relationship of CSR activities and translates it into a collection of transactions consisting of stakeholder objects.”* |
|  |  |
| **Reviewer #2** |  |
| **Reviewer’s Comments** | **Authors’ Responses** |
| 2.1 In my view, authors can also use the lens of legitimacy theory to further support their argument regarding CSR implementation in response of stakeholders’ management/relationship in the context of construction industry. For legitimacy theory, authors can refer the following studies, among others.  • Di Vaio, A., Varriale, L., Di Gregorio, A., & Adomako, S. (2022). Corporate social performance and non‐financial reporting in the cruise industry: Paving the way towards UN Agenda 2030. Corporate Social Responsibility and Environmental Management, 29(6), 1931-1953. • Velte, P. (2022). Meta-analyses on corporate social responsibility (CSR): a literature review. Management Review Quarterly, 72(3), 627-675. | Thanks to the reviewer for your suggestions. We added the legitimacy theory perspective in **[2.3 CSR stakeholder relationship in construction]** as a way of explaining potential stakeholder relationships. And we have added the papers recommended by the reviewer.  **Please see lines 168-180:**  *“Stakeholder analysis, a part of the stakeholder management process [**53**], involves companies gathering information on stakeholder issues to manage changing interests [**54**]. Institutions that shape incentives and legitimacy affect the social behavior of companies. Stakeholders gain legitimacy and power from these institutions, impacting the company’s CSR strategy. Additionally, legitimacy theory presumes a social contract between organizations and society [**55**]. In the context of CSR, supporting effective tools to convey the organization’s legitimizing actions and gaining legitimacy is considered to be an important resource for the organization [**56**]. “Stakeholder legitimacy” is defined when stakeholders claim legitimacy in organizations, addressing their demands [**57**]. This interplay between legitimacy and the stakeholder perspective fosters dialogue between organizations and stakeholders [**58**]. Leveraging legitimacy and understanding stakeholder salience helps organizations manage multi-stakeholder relationships based on the priority given to stakeholder advocacy.”* |
| 2.2 In the literature review section, I would suggest authors to add hypothesis for each link, for instance, regarding stakeholder-based view on CSR and stakeholder relationship management. | Thanks the reviewer for your suggestions. We re-wrote **[2. Literature Review]** and presented the three hypotheses of this manuscript in this part：  *“****Hypothesis (H_1_)****: Companies prioritize the implementing of key CSR activities and leverage their associated relationships in the fulfillment process.”*  *“****Hypothesis (H_2_)****: Companies prioritize meeting the needs of key stakeholders.”*  *“****Hypothesis (H_3_)****: There are associated relationships between stakeholders.”*  **Please see [2. Literature Review]** |
| 2.3 In the abstract, authors mentioned that “the results show that the SCSR-2M can be composed of three key stakeholders, namely government, partners, NGOs and media.” I think these are four stakeholders not three or authors might consider both NGOs and media as one stakeholder. Confirm the same in rest of the manuscript. | Thank you for your comments. We base our review on the CSR report, as well as related literature, such as Lin et al., (2019), which suggests that “the main impact of NGOs is to use their expertise and knowledge to lobby or make appeals to persuade governments or corporations to respond to social responsibility issues. The expertise and public relations that NGOs possess are important resources for companies concerned about their social and environmental image. And the media are also companies concerned about their social and environmental image.” Meanwhile, according to a typical construction company CSR report, such as China Railway Construction Corporation Limited’s 2020 Corporate Social Responsibility Report, it is clearly stated that “Individuals, groups (including NGOs) and the media who are interested in the company expect the company to be a responsible enterprise and to achieve sustainable development, which includes disclosing information in accordance with the law, press conferences, and news coverage and publicity”.  Therefore, this study places NGOs and the media in a category of stakeholder groups, both of whom are external regulators of the firm but do not have a direct interest in the firm. In future research, we can further refine more stakeholders, including subcontractors, designers, supervisors, etc. among the partners, as well as NGOs and media.  Reference:  [1] Lin X, Mckenna B, Ho C M F, Shen G Q P. Stakeholders' influence strategies on social responsibility implementation in construction projects. Journal of Cleaner Production. 2019; 235: 348-358. |
| 2.4 Finally, the paper should go through English proof editing. The paper is not very clear to the reader. | Thanks to the reviewer’s suggestions, we have thoroughly edited and enhanced the language of the manuscript. |
|  |  |
| **Reviewer #3** |  |
| **Reviewer’s Comments** | **Authors’ Responses** |
| 3.1 However, there is insufficient analysis of the data results and the reasons for their formation, especially in section 5. Findings and Discussion of the paper. The author used nearly 60 literatures to illustrate what? Is it just that the results are consistent with previous research? If so, what is the significance of this article? And the author stated in the abstract that "this article is the earliest effort on this topic". I guess what readers most want to see is the content of this article that is different from existing literatures, and the suggested strategies that are consistent with the data results. | Thanks for your suggestions. On the one hand, we pointed out the similarities between the results of this study and those obtained from other studies, showing that the results of our study are scientific and reliable to a certain extent and can be confirmed by other studies. On the other hand, we further explained the results of this study in the light of the reviewer’s suggestions in conjunction with **[2.3 CSR stakeholder relationship in construction]**.  For example, when **[5.1 Associations of CSR activities in the construction industry]** explains CSR activities associations:  **Please see lines 447-465:**  ***“****CSR implementation is not isolated or scattered, but associated through a combined method, and integrates social, environmental, and economic factors into corporate strategy and practices, resulting in cost reductions, enhanced innovation, and strengthened environmental protection [**77**]. This balanced approach fosters long-term sustainability by considering the values associated with social, environmental, and economic aspects [**78**], which confirms* ***H_1_****. Because CSR consumes resources, construction firms typically expand their resources to implement one or more CSR activities. In order to generate profit, the sum of the costs of acquiring these resources must be less than the total revenue that these resources together generate. Under the condition of given resources, CSR activities are linked to limited capabilities and resources, and information such as the cost of implementing CSR and the scarcity of resources are carefully analyzed. Selective CSR activities not only create CSR differentiation and competitiveness, but also generate mutual benefits for stakeholders. To optimize resource utilization, enterprises should adopt a combined CSR performance approach, as supported by existing research and depicted in Fig 4 and Fig 5. For example, when an enterprise serves the community, it can combine community greening, caring, publicity and other activities to reduce the number of repetitions and the use of resources; enterprises can reduce emissions and produce more environmentally friendly products due to consumers’ environmental demands.****”***  The manuscript explains the key stakeholders of a construction company and their characteristics in 5.2.  **Please see [5.2 Key stakeholder involved in construction].**  The manuscript explains in 5.3 the possible reasons for the emergence of stakeholder associations and suggests appropriate management approaches based on the implied relationships.  **Please see [5.3 Strategies for stakeholder relationship management in CSR].** |

[The End]
